# Bayesian-Based Pharmacokinetic Framework Integrated with Therapeutic Drug Monitoring for Assessing Adherence to Antiseizure Medications: A Clinical Trial Simulation Study

**DOI:** 10.2196/77917

**Published:** 2026-01-02

**Authors:** Xiao-Qin Liu, Zi-Ran Li, Wei-Wei Lin, Juan Wang, Fu-Qing Gu, Jun-Jie Ding, Zheng Jiao

**Affiliations:** 1 Department of Pharmacy, Shanghai Chest Hospital, Shanghai Jiao Tong University School of Medicine, 241 West Huaihai Road, Shanghai, 200030, China, 86 15502125571; 2 Department of Bioengineering & Therapeutic Sciences, University of California, San Francisco, San Francisco, CA, United States; 3 Department of Pharmacy, The First Affiliated Hospital of Fujian Medical University, Fuzhou, Fujian, China; 4 Center for Tropical Disease and Global Health, Nuffield Department of Clinical Medicine, University of Oxford, Oxford, United Kingdom

**Keywords:** antiseizure medications, medication adherence, therapeutic drug monitoring, Bayesian theory, population pharmacokinetics

## Abstract

**Background:**

Adherence to antiseizure medications (ASMs) is a cornerstone of effective epilepsy management. However, current consensus guidelines for assessing medication adherence via therapeutic drug monitoring (TDM) may neglect individual patient characteristics, thereby compromising the accuracy of adherence assessments.

**Objective:**

This study proposed an innovative Bayesian–based pharmacokinetic (PK) framework integrated with TDM data to address the above limitations, with a focus on 14 widely prescribed ASMs, including brivaracetam, carbamazepine, clobazam, eslicarbazepine acetate, lacosamide, lamotrigine, levetiracetam, oxcarbazepine, perampanel, phenobarbital, topiramate, valproic acid, vigabatrin, and zonisamide.

**Methods:**

Comprehensive clinical trial simulations were conducted to investigate the PK of ASMs in patients with epilepsy under conditions of full adherence and various nonadherent dosing behaviors, including omission of the last dose and consecutive missed doses. Bayesian posterior probabilities of these dosing behaviors were derived by integrating validated population PK models, individual patient demographics (eg, age, weight, creatinine clearance), dosing history, prior adherence probabilities and TDM measurements. Additionally, the influence of covariates on assessment outcomes was systematically evaluated.

**Results:**

The Bayesian-based PK approach demonstrated robust discriminative ability. Under idealized simulation conditions with minimized variabilities, the approach achieved accurate retrodiction of the last 1 or 2 doses across all 14 ASMs and partial retrodiction of extended nonadherence trajectories for 6 ASMs. Concentration thresholds for adherence classification varied significantly across drugs and are influenced by patient-specific factors, comedications, formulation, sampling time, and prior probability. To translate these insights into practice, an adaptable web-based dashboard was developed using the *shiny* package in R software to enable precise and real-time assessments of medication adherence.

**Conclusions:**

This study establishes a Bayesian-based PK approach to enhance the assessment of ASMs adherence. This approach facilitates a paradigm shift from population-based management to patient-specific adherence profiling, offering a practical methodology for the precise evaluation of medication-taking behaviors.

## Introduction

Epilepsy is the second most common neurological disease globally. Antiseizure medications (ASMs) represent the cornerstone of treatment for epilepsy [1,2], with long-term medication adherence being critical to achieving successful therapeutic outcomes [3]. However, adherence to ASMs among people with epilepsy is often suboptimal [4-6], which is strongly associated with a range of adverse clinical outcomes, including increased mortality, heightened morbidity, greater health care utilization, and substantial economic burden [7,8]. Therefore, when evaluating treatment failures, it is imperative for health care providers to comprehensively assess patients’ adherence, to identify underlying issues and provide tailored support to improve seizure control and treatment efficacy.

In clinical practice, self-reported adherence is inherently subjective and prone to bias [9], in contrast to therapeutic drug monitoring (TDM), which offers a more objective measure of recent medication-taking behaviors [10,11]. Accurate TDM interpretation is straightforward in some situations, such as a notably low drug concentration suggesting nonadherence. However, it becomes much more complex in other cases due to various intrinsic and extrinsic confounders that affect drug concentrations, including organ function, drug-drug interactions and dosing intervals.

The Consensus Guidelines for TDM in Neuropsycho-pharmacology: 2017 update [12] (hereafter referred to as the 2017 Guidelines) provide reference ranges for commonly used ASMs, which are specifically tailored for steady-state trough concentrations (C_0_) in adult patients undergoing monotherapy. The reference ranges for each ASM were determined by multiplying the daily dose by dose-related concentration factors, and then could be used to help identify nonadherence [12]. However, the reference ranges were based on average pharmacokinetic (PK) parameters from an adult population, and do not account for key subpopulations, such as pediatric, geriatric and pregnant patients, who exhibit clinically significant PK differences [13-15].

PK modeling and simulation approaches have been successfully used to evaluate the impact of medication nonadherence [16,17], and to design remedial dosing strategies for missed or delayed doses [16,18]. When combined with Bayesian principle, this methodology offers a powerful framework for integrating individual TDM data with population PK models [19,20]. By leveraging this approach, it becomes possible to infer posterior probabilities of different dosing patterns, thereby enabling a more refined and quantitative assessment of medication-taking behavior.

In light of the above, this study aims to characterize medication adherence patterns to ASMs using TDM measurements and a Bayesian-based PK approach. Additionally, a user-friendly dashboard is developed to offer health care providers an intuitive, practical tool for assessing individual adherence levels, thereby optimizing ASM therapy and improving the treatment outcomes of ASMs.

## Method

### Ethical Considerations

As this study exclusively used computational modeling and simulation techniques without involving direct human subject participation or personal data collection, it is exempt from institutional review board approval requirements in accordance with international ethical guidelines.

### Rationale

When patients fully adhere to their medication regimens, the drug concentration fluctuates in a predictable manner. However, if patients miss any of their doses, the drug concentration will gradually decline to a suboptimal level, which may ultimately result in treatment failure. The differences in the probability distribution of drug concentration provides a valuable reference for differentiating between adherence to the prescribed medication and nonadherence.

In this study, the Bayesian-based PK approach, calculating the posterior probability of special dosing events, was used to assess medication adherence. The principle of the Bayesian approach is as follows [19]: given the probability of the occurrence of a specific scenario (ie, the prior probability, $P(\omega_{j})$) and the probability of a particular drug concentration at a given scenario $\omega_{j}$ (ie, conditional probability, $P\left(C|\omega_{j}\right)$) , the probability of the scenario at a given drug concentration (ie, posterior probability,$P(\omega_{j}\left|C\right)$) can be estimated, as presented in Equation 1.

       (1) $$P(\omega_{j}|C)=\frac{P(\omega_{j})\times P(C|\omega_{j})}{P(C)}$$

Where P(C) is the full probability and could be calculated with Equation 2.

      (2) $$P(C)=\sum_{j}P(\omega_{j})\times P(C|\omega_{j})$$

The prior probability $P\left(\omega_{j}\right)$ refers to the pre-existing or baseline probability estimate of a patient’s likelihood to adhere to a prescribed medication regimen before any new, specific data related to that individual patient’s adherence behavior in the current treatment course is considered. The conditional probability $P(C|\omega_{j})$ is calculated using Monte Carlo simulations based on population PK. Once these probabilities have been obtained, the posterior probability $P(\omega_{j}\left|C\right)$ of each individual scenario is calculated using Equations 1; 2. The scenario with the highest posterior probability is considered the most likely to occur, while the one with the lowest posterior probability is deemed the least probable.

**Figure 1.**
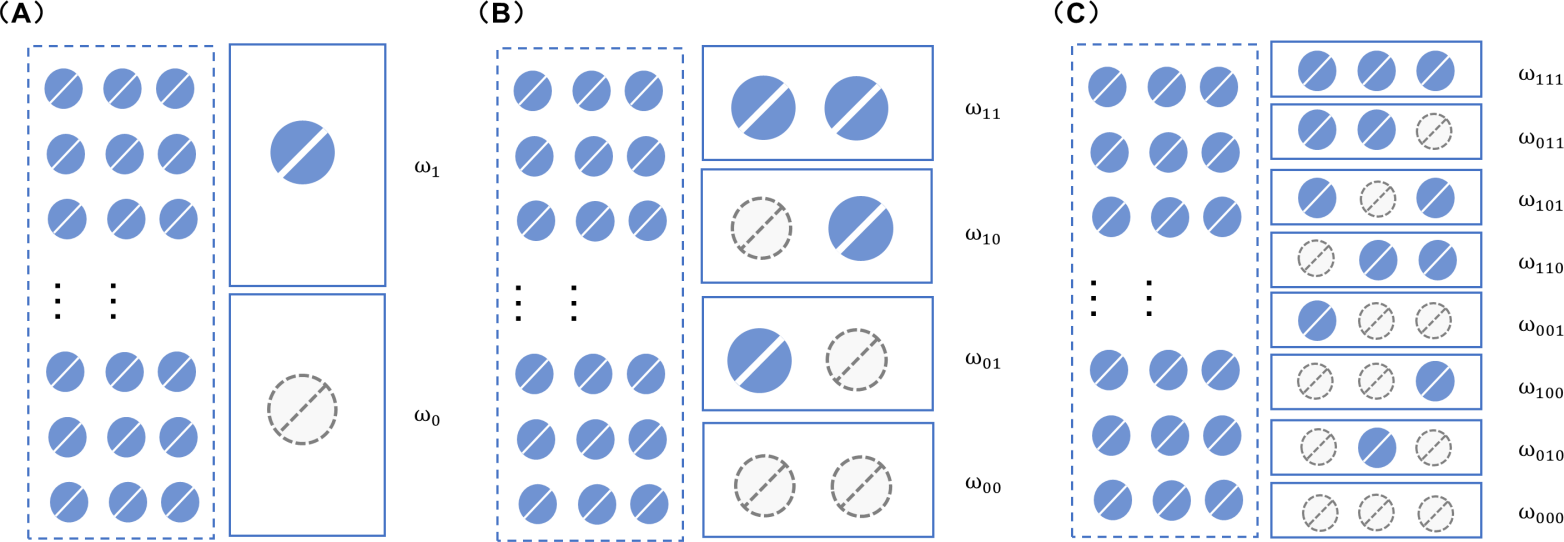
The dosing scenarios when the most recent one (A), two (B) or three (C) dosing events are considered. ω: medication-taking behavior, where the first, second and third digit after ω indicates the most recent one, two and three medication-taking events prior to sampling, respectively, where 1 indicates dose taken and 0 indicates dose missed.

In our study, the dosing event scenario is defined by whether patients adhere to or miss their scheduled doses. There are 2^n^ possible scenarios when considering the last *n* dosing events prior to sampling. For instance, as depicted in Figure 1A, there are two scenarios ($\omega_{0}$ and $\omega_{1}$) when considering the last dosing instance. This can be expanded to four scenarios ($\omega_{00}$, $\omega_{01}$, $\omega_{10}$, and $\omega_{11}$) when the last two dosing instances are considered (Figure 1B), and eight scenarios ($\omega_{000}$, $\omega_{010}$, $\omega_{100}$, $\omega_{001}$, $\omega_{110}$, $\omega_{011}$, $\omega_{101}$, $\omega_{111}$) when the last three dosing events are taken into account (Figure 1C).

**Figure 2.**
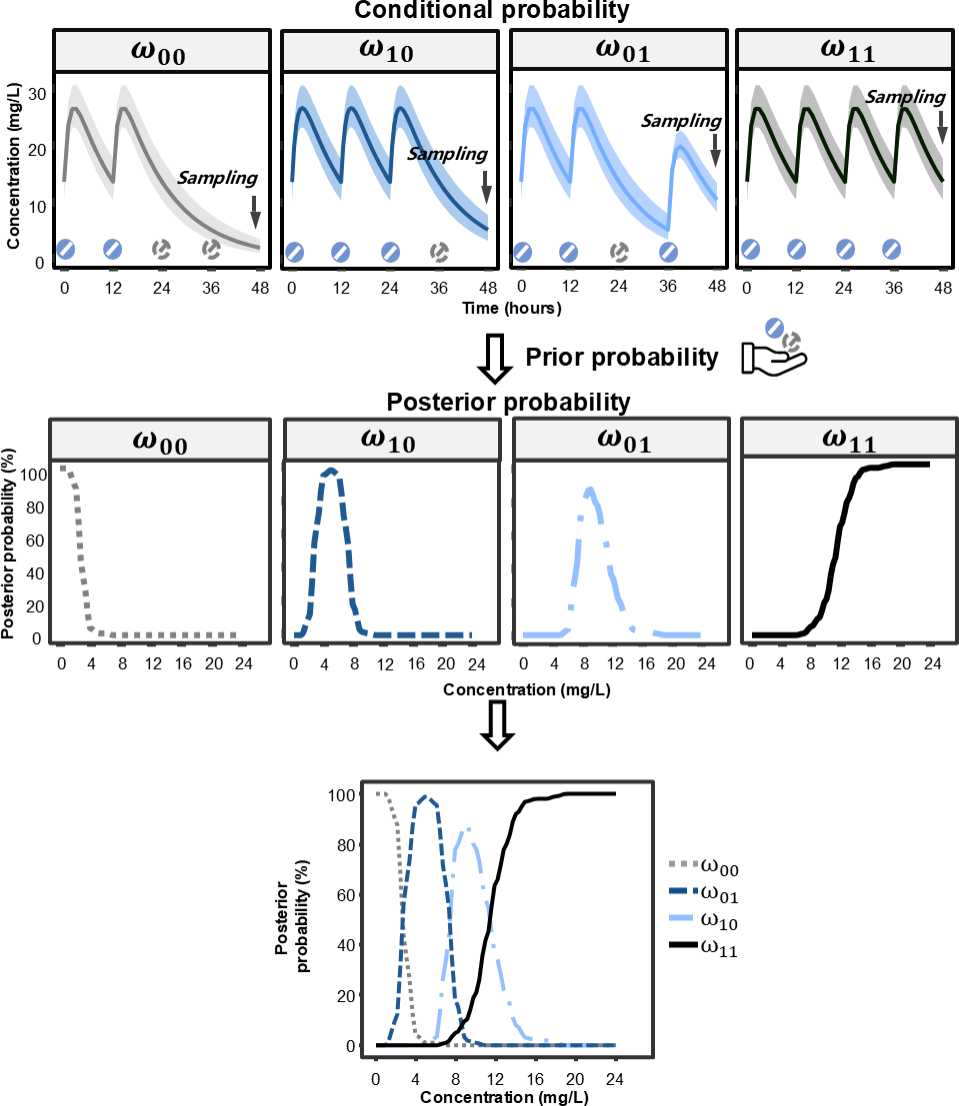
The workflow of adherence assessment by therapeutic drug monitoring with Bayesian-based pharmacokinetic approach.

The workflow of adherence assessment is graphically represented with Figure 2, using the example of a 70 kg adult patient receiving oxcarbazepine 300 mg every 12 hours (q12h) and reached steady state. C_0_ of oxcarbazepine was used to infer the patient’s dosing behavior over the last two dosing intervals. When the C_0_ approaches zero, the posterior probability of at least one missed dose is high. As C_0_ increases, this probability decreases, while the posterior probability of complete adherence rises correspondingly, eventually approaching 100%.

The C_0_ value at which the posterior probabilities of two distinct dosing scenarios are equal, can be derived from Equations 1; 2, serving as a threshold to discriminate between these scenarios. As depicted in Figure 2, the posterior probabilities of missing two doses ($\omega_{00}$) and missing only the last dose while having taken the second last dose ($\omega_{01})$ are equal when the C_0_ is approximately 3 mg/L. When C_0_ is less than 3 mg/L, the probability of $\omega_{00}$ is the highest. Similarly, the C_0_ range maps to the most probable scenario as follows: 3‐7.5 mg/L to $\omega_{01}$, 7.5‐11.5 mg/L to missing the second-last dose but taking the last dose ($\omega_{10}$) , and levels above 11.5 mg/L to taking both of the last two doses ${(\omega }_{11})$.

**Figure 3.**
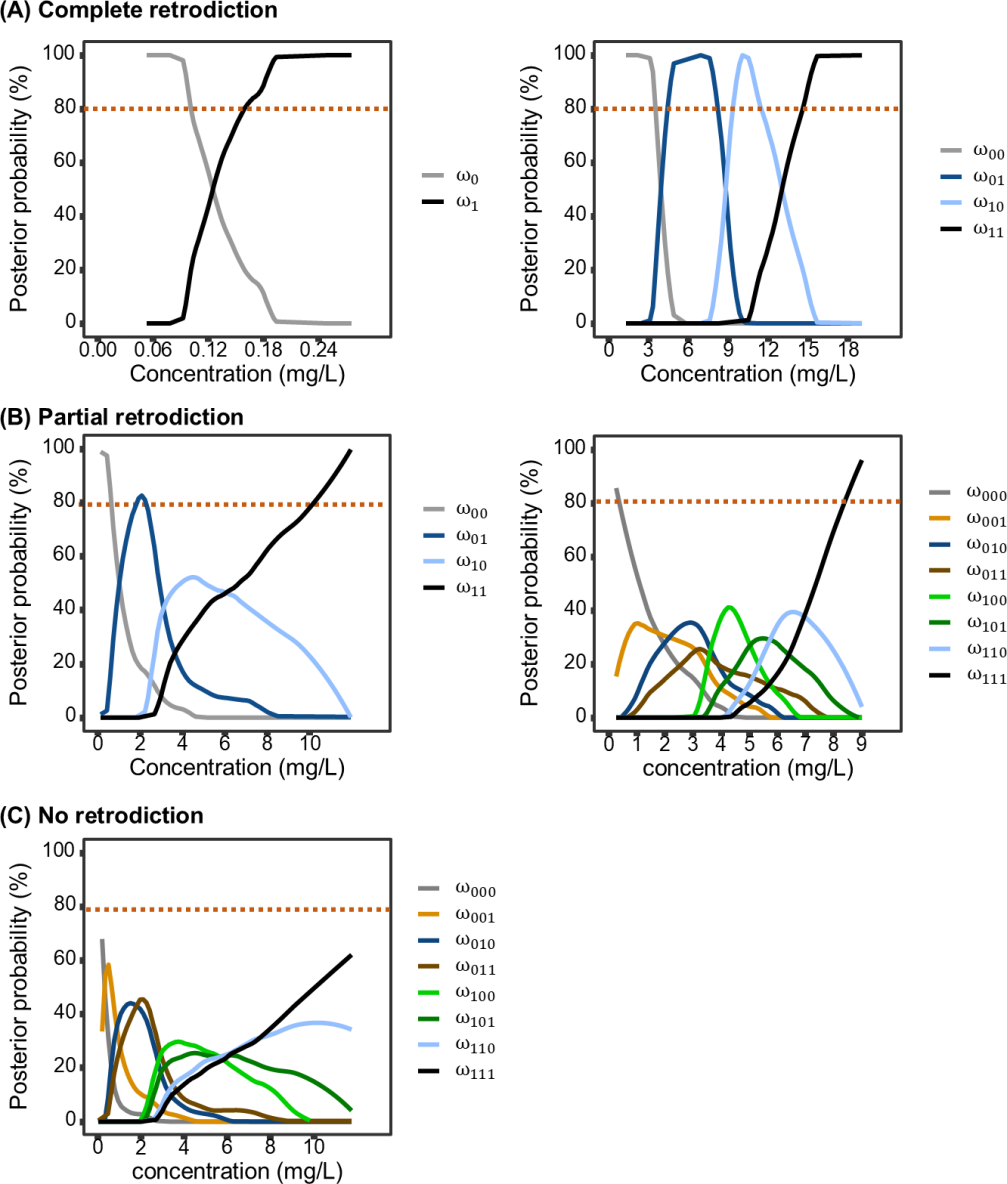
Illustration of retrodiction types based on posterior probabilities of dosing events prior to sampling. (A) complete retrodiction: the maximum posterior probabilities of all dosing events are ≥80%; (B) partial retrodiction: only events which are fully adherent and fully nonadherent have a maximum posterior probability ≥80%; and (C) no retrodiction: only one or no dosing events have a maximum posterior probability ≥80%.

In this case, all dosing events could have a maximum posterior probability exceeding 80%, which was defined as complete retrodiction (Figure 3A). If only one or none dosing events had a maximum posterior probability exceeding 80%, it was defined as no retrodiction (Figure 3C). Other cases were defined as partial retrodiction (Figure 3B).

### Population Pharmacokinetic Characteristics

To characterize the conditional probability associated with each dosing scenario, a systematic literature search was conducted in PubMed and Embase to collate available population PK parameters for ASMs across various formulations, including conventional tablets, oral solutions, suspensions, syrups, and extended-release (ER) formulations. In our previous study [18], population PK models for 10 commonly used ASMs were identified up to March 31, 2022, including carbamazepine, clobazam, eslicarbazepine acetate, lamotrigine, levetiracetam, oxcarbazepine, phenobarbital, topiramate, valproic acid, and zonisamide. An update search was then performed up to November 30, 2024. Additionally, the population PK characteristics of brivaracetam, lacosamide, perampanel and vigabatrin were also incorporated in the study. Details of the literature review were summarized in Multimedia Appendix 1.

### Assessment of Adherence

During the adherence assessment, pediatric patients (aged 8 y, weighing 25 kg, and measuring 127 cm), adult patients (aged 40 y, weighing 70 kg, and measuring 180 cm), and pregnant women (aged 25 y, weighing 70 kg, measuring 160 cm, 30 wk pregnant) taking conventional tablet of ASMs were selected as typical patients. All typical patients had normal renal and liver function and were not taking any concomitant medications.

The evaluated dosing behaviors included the administration of the last one, two, and three doses prior to sampling at steady state. Sampling was conducted immediately before the subsequent dose. As for prior probability, it was assumed that each scenario had an equal chance of occurring. Specifically, this implies a probability of 50% for each scenario when considering only the last dosing behavior, 25% when considering the last two dosing behaviors, and 12.5% when considering the last three dosing behaviors.

For the Monte Carlo simulations, the parameters were fixed according to the final reported values, except for the residual unexplained variability (RUV) to obtain the “true” concentration-time profiles under various nonadherence scenarios. Consequently, RUV was set to negligible levels [21]—specifically 0.01 mg/L for additive error and 0.1% for proportional error—to minimize noise in the study. A total of 40,000 virtual patients were generated for each scenario. The Monte Carlo simulations were conducted using R programming (version 4.2.2; R Foundation for Statistical Computing) with the *rxode2* package (version 2.1.2). The results were plotted using the *ggplot2* package (version 3.5.1).

### Evaluation of Critical Factors Affecting Adherence Assessment

The factors reported to significantly influence the PK of ASMs were investigated for their impact on adherence assessment, including renal function (estimated glomerular filtration rate, eGFR: 30, 60 and 90 mL/min/1.73m^2^) and concomitant medications. Additionally, the effect of formulation (extending dosing interval to every 24 h for ER formulation), sampling time (2 h earlier or later), and prior probabilities (10%, 30%, 50%, 70%, and 90%) on medication adherence was also tested. The impact of these factors was assessed from two perspectives: the first was the ability to retrodict the number of the last scheduled doses, and the second was their influence on the concentration threshold used to distinguish between nonadherence patterns.

### Development of Web-Based Dashboard

To facilitate quick calculation, an interactive online dashboard was developed to assess ASMs’ medication adherence, informed by TDM results, and individual characteristics that were determined as significant factors on PK parameters in the included models. This tool was built using *rxode2* (version 2.1.2), *ggplot2* (version 3.5.1), and *shiny* (version 1.8.1.1) within the R framework (version 4.2.2; R Foundation for Statistical Computing).

## Results

### Population Pharmacokinetic Characteristics

A total of 23 population PK models encompassing 14 ASMs were ultimately included in the analysis [22-44]. Among these, models for adult [22-25,27-29,31,33,35,36,38,40,42] and pediatric patients [22,23,25-30,32,34,36,37,39,44] were available, while models specific to pregnant women were only available for lamotrigine [41] and levetiracetam [43]. Since age was consistently identified as a significant covariate for PK parameters in adults, elderly patients were thus grouped with the adult population. Models characterizing multiple formulations were identified for eslicarbazepine acetate [34], lamotrigine [36] and valproic acid [30,42]. Details of the identification of literature, included studies, and the final parameter estimates used in the analysis were comprehensively summarized in Figures S1-S9 in Multimedia Appendix 1 and Tables S1-S2 in Multimedia Appendix 1.

### Assessment of Adherence

**Figure 4.**
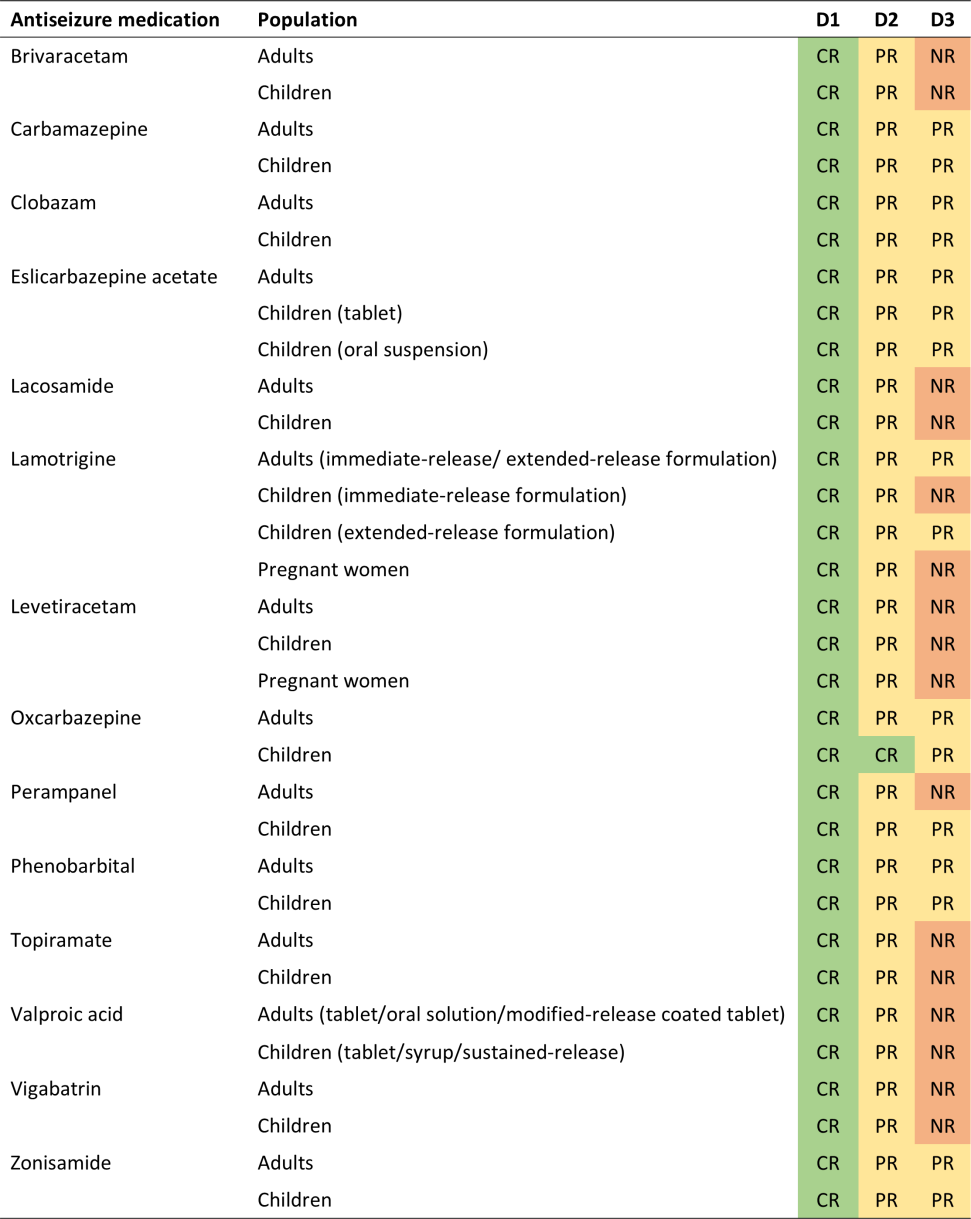
The ability to retrodict the last one, two and three dosing behaviors prior to sampling for typical patients in theoretical condition when minimizing residual unexplained variabilities. D1: the last dosing behavior; D2: the last two dosing behaviors; D3: the last three dosing behaviors; CR, complete retrodiction, which is defined as when the maximum posterior probabilities of all dosing events are ≥80%; PR, partial retrodiction, which is defined as when only events which are fully adherent and fully nonadherent ( have a maximum posterior probability ≥80%; NR: no retrodiction, which is defined as when only one or no dosing events have a maximum posterior probability ≥80%. Adults: aged 40 y, weighing 70 kg, and measuring 180 cm; children: aged 8 y, weighing 25 kg, and measuring 127 cm; pregnant women: aged 25 y, weighing 70 kg, measuring 160 cm, and being 30 weeks pregnant. Residual unexplained variabilities were minimized by defining additive error as 0.01 mg/L and proportional error as 0.1%.

The posterior probabilities of various dosing behaviors when considering the last one, two, and three dosing behaviors for each ASM are detailed in Figure S10-S23 in Multimedia Appendix 1. Figure 4 demonstrates the ability to retrodict the number of the last scheduled doses for each ASM in typical patients under conditions of minimized RUV. Results indicated that when investigating the most recent dosing behavior, all ASMs can be fully retrodicted. Regarding the scenarios involving the last two dosing behaviors, complete retrodiction was achievable only for oxcarbazepine in pediatric patients, whereas other ASMs were partially retrodicted. When extending to the last three dosing behaviors, no ASMs can be fully retrodicted, and only carbamazepine, clobazam, eslicarbazepine acetate, oxcarbazepine, phenobarbital and zonisamide could be partially retrodicted across all investigated population. From a pharmacokinetic perspective, ASMs with higher clearance are eliminated rapidly, thereby diminishing the concentration “signal” necessary to distinguish earlier dosing events. Furthermore, the traceability may vary in clinical scenarios where patient characteristics significantly deviate from the typical population.

### Impact of Critical Factors on Adherence Assessment

It has been reported that renal function affects the apparent clearance (CL/F) of eslicarbazepine acetate [33], levetiracetam [24], oxcarbazepine [38] and vigabatrin [28]. As renal function decreases, there is no significant effect on the identification of nonadherence patterns. The effect of renal function on levetiracetam is illustrated in Figure S24 in Multimedia Appendix 1, with levetiracetam, oxcarbazepine, and vigabatrin demonstrating similar trends.

Pregnancy enhances the clearance of lamotrigine and levetiracetam, leading to lower systemic drug exposure and, consequently, may decrease the concentration thresholds (Figure S15-S16 in Multimedia Appendix 1). Similarly, pediatric patients show higher clearance per body weight compared with adults, leading to the decreased concentration thresholds (Figure S10-S23 in Multimedia Appendix 1).

The effects of concomitant inducers and inhibitors were also evaluated. Administration of inducers or inhibitors did not alter the fundamental distinguishability of nonadherence patterns but shifted the concentration threshold required for their discrimination. Specifically, the threshold was lowered by inducers and raised by inhibitors. The magnitude of these adjustments varied substantially across ASMs (Figure S25 in Multimedia Appendix 1). For instance, in a typical adult patient taking lamotrigine, coadministration with enzyme inducers (eg, carbamazepine, phenobarbital) lowered the threshold by approximately 67%, whereas the inhibitors valproic acid elevated it by approximately 83%. The effect on topiramate was less pronounced.

The impact of formulation on adherence assessment was evaluated for eslicarbazepine acetate, lamotrigine and valproic acid. At equivalent total daily doses, ER formulations with prolonged dosing intervals enhanced the discriminative capacity for dosing behaviors compared to immediate-release (IR) or other oral formulations requiring more frequent administration (Figure S15, S21 in Multimedia Appendix 1). In contrast, formulations such as oral suspensions and syrups exhibited minimal impact on the assessment (Figure S13, S21 in Multimedia Appendix 1).

Sampling time also influences adherence assessment (Figure S26 in Multimedia Appendix 1). Sampling 2 hours earlier or later than the scheduled time does not significantly influence the distinguishability of nonadherence patterns. However, compared to sampling just before administration, the concentration threshold for distinguishing nonadherence patterns increases when sampling is done earlier and decreases when sampling is done later. The magnitude of this change varies among different ASMs.

The impacts of prior probabilities on adherence assessment were also evaluated. The results indicated that prior probabilities could not only significantly affect the distinguishability of the nonadherence dosing scenarios, but also notably alter the concentration threshold for distinguishability (Figure S27 in Multimedia Appendix 1). The magnitude of the concentration threshold change was found to be dependent on the type of ASMs.

### Application of Web-Based Dashboard

A web-based dashboard for assessing medication adherence has been developed and is freely accessible online [45]. After inputting the type of ASMs, patient characteristics (age, body weight, height, gender), scheduled dosing regimens, sampling time, TDM data, and prior probabilities for each scenario, the system estimates the posterior probabilities of each dosing scenario and plots them against the drug concentration. RUV are initialized with literature-reported values (as listed in Table S2 in Multimedia Appendix 1) when requiring consideration, but remain user-adjustable to accommodate specific clinical situations, thereby enabling the precise identification of medication adherence patterns.

**Figure 5.**
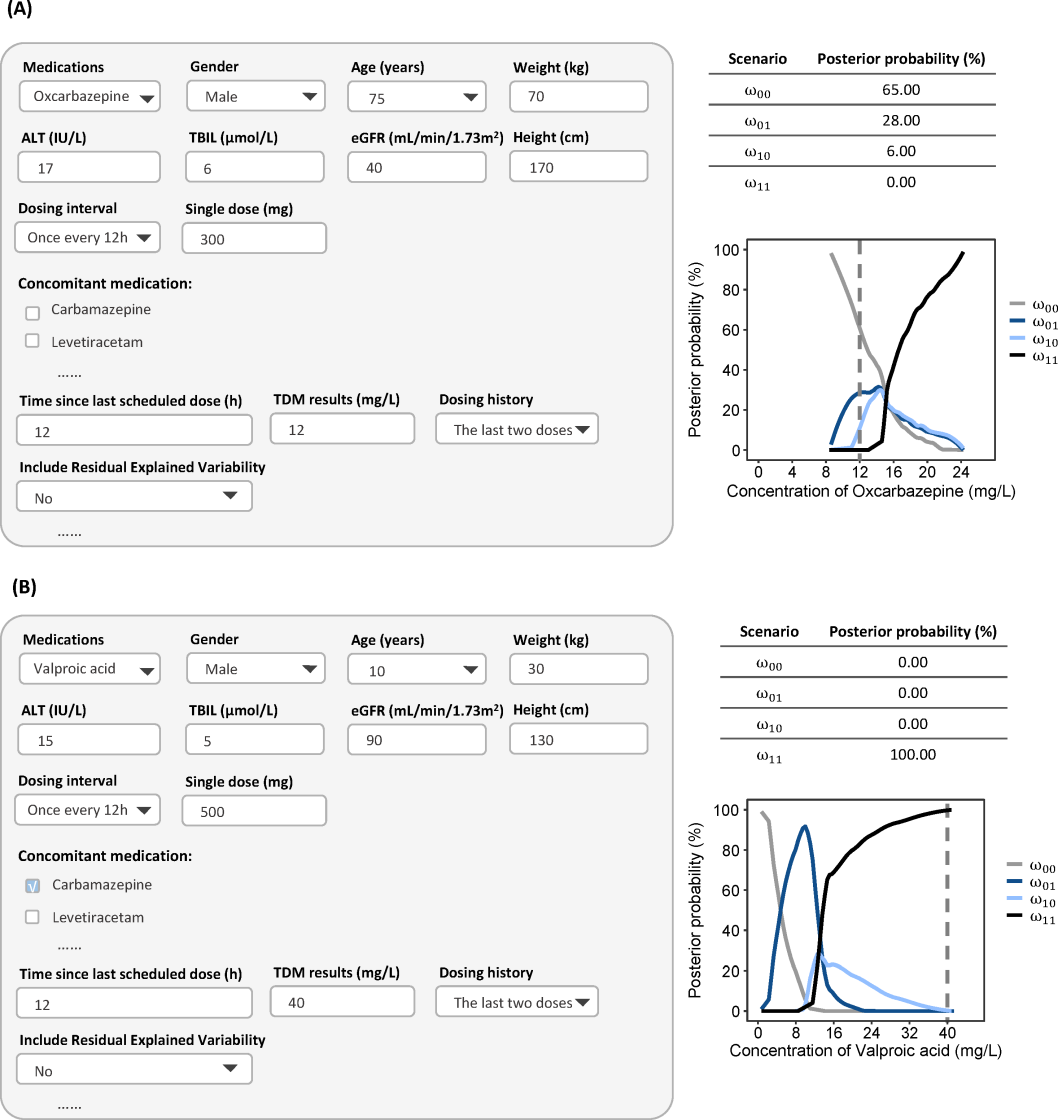
Screenshot of the dashboard for adherence assessment. (A) Elderly patient, 75 years old, weighing 70 kg, measuring 180 cm, eGFR 40 mL/min/1.73m^2^, taking oxcarbazepine 300 mg q12h; (B) pediatric patient, 10 years old, weighing 30 kg, measuring 130 cm, taking valproic acid 500 mg q12h and carbamazepine 100 mg q12h. ɷ_00_: missing two continuous doses before sampling; ɷ_01_, missing the second-to-last dose but taking the last dose; ɷ_10_, missing the last dose but taking the second-to-last dose; ɷ_11_*,* taking all doses*.*

Figure 5A presents a case of a 75-year-old male (70 kg) with epilepsy and impaired renal function (eGFR 40 mL/min/1.73m^2^) who had remained seizure-free for over 3 years on oxcarbazepine 300 mg q12h. Following a recent increase in seizure frequency, TDM was performed to assess potential nonadherence. The measured C_0_ of oxcarbazepine was 12 mg/L, which lies within the conventional therapeutic range of 10‐35 mg/L and aligns with the recommended range of 6‐24 mg/L as per the 2017 guidelines [12]. However, model-based estimates from the dashboard indicated a nearly negligible probability of full adherence and a high probability of having missed at least one dose. The posterior probability of full adherence remained at 0%, regardless of whether the prior probability was set as low as 1% (suggesting poor adherence) or as high as 99% (denoting high adherence) (Figure S28 in Multimedia Appendix 1), demonstrating the minimal influence of the prior in this case. By contrast, increasing the RUV from 0.1% to 30% increased the posterior probability of full adherence from 0% to 56% (Figure S29 in Multimedia Appendix 1), underscoring the substantial impact of RUV on the adherence assessment in this case.

Figure 5B illustrates another case of a 10-year-old (30 kg) boy with epilepsy and normal renal/hepatic function, treated with valproic acid tablet 500 mg and carbamazepine 150 mg q12h. His measured C_0_ of valproic acid was 40 mg/L, below both the conventional therapeutic range (50‐100 mg/L) and the 2017 guideline-recommended range (62.2‐134.8 mg/L). Although subtherapeutic concentrations initially raised suspicion of nonadherence, model-based analysis estimated a probability of full adherence exceeding 80%, suggesting that the low valproic acid concentration likely resulted from carbamazepine-induced metabolic induction rather than missed doses. Varying the prior probability of adherence from 1% to 99% had minimal impact on this conclusion (Figure S30 in Multimedia Appendix 1). Similarly, increasing RUV from 0.1% to 30% altered the posterior probability of full adherence by only 5% (Figure S31 in Multimedia Appendix 1), demonstrating the robustness of the assessment against RUV variation in this clinical scenario.

## Discussion

### Principal Findings

This study is the first to introduce a clinical framework to investigate the role of TDM in assessing medication adherence for 14 commonly used ASMs among diverse patients. By integrating Bayesian theory with population PK, we demonstrated that routine TDM, when combined with clinical factors, enables quantitative retrodiction of recent medication-taking behaviors for all investigated ASMs. The Bayesian-based PK framework can also be easily available through the open-access dashboard developed in this study.

### Comparison to Prior Work

The 2017 Guideline established reference ranges encompassing approximately 66% of patients for commonly used ASMs, primarily derived from PK data obtained in adult patients receiving monotherapy [12]. While clinically useful, these population-derived thresholds have limited generalizability to special populations with distinct PK profiles. In contrast, the model-informed algorithm developed in this study enables a more personalized assessment of medication adherence. This approach explicitly accounts for patient-specific factors including age (eg, pediatric and geriatric populations), pregnancy status, renal and hepatic function, concomitant use of enzyme inducers or inhibitors, formulation characteristics. It thereby provides a refined framework for evaluating medication-taking behavior across diverse clinical scenarios.

### Interpretation of the Findings

We identified multiple critical factors that influence the adherence assessment, including intrinsic factors (physiological differences, concomitant medication, renal function, formulation , etc) and extrinsic factors (prior probability, RUV, etc).

The influence of intrinsic factors on adherence evaluation is primarily mediated through alterations in PK parameters, most notably systemic drug clearance. Enhanced drug clearance, commonly observed in pediatric and pregnant patients, as well as in those receiving enzyme inducers, reduces both the ability to differentiate adherence patterns and the corresponding concentration thresholds. In pediatric populations, the higher clearance per body weight results from ongoing organ maturation, larger organ size-to-body weight ratios, and increased metabolic enzyme activity [15,36,46]. In pregnant women, elevated clearance arises from increased cardiac output, enhanced renal blood flow, and hormonally mediated induction of metabolic pathways [14,41,47,48]. Conversely, reduced clearance, frequently encountered in patients with renal impairment or those receiving enzyme inhibitors, may improve differentiation ability or raise the concentration thresholds required for pattern discrimination.

Despite the increasing use of ER formulations of ASMs, conventional formulations continue to account for a substantial proportion of prescriptions due to their lower cost and wider availability [49,50]. Consequently, population PK studies have more frequently characterized conventional formulations. Based on the limited population PK data available for ER ASMs, our findings suggest that ER formulations may improve the differentiation of adherence behaviors, a finding attributable to the extended dosing interval (eg, from 12 to 24 h) and the resulting concentration-time fluctuation.

Prior probability is essential for estimating the posterior probability of dosing behaviors. In this study, we adopted an equiprobable prior probability to reflect a state of maximum uncertainty before considering the evidence (TDM measurements). This represents a conventional and conservative strategy in Bayesian modeling when reliable, specific prior knowledge is unavailable [19,51]. In real clinical settings, the prior probability can be informed by pharmacy refill data or population-average adherence estimates. When individual-level data are absent, population-based priors derived from patients with comparable covariates (eg, age, comorbidities, and socioeconomic status) may be applied. Although the impact of the prior was limited in the cases illustrated in Figure 5, its influence on adherence assessment can be substantial and depends on both the specific ASM and TDM measurements. Consequently, the ability for user-defined priors implemented in the dashboard remains highly valuable.

RUV in population PK analysis captures unexplained stochastic variations, including assay error, sampling inaccuracies, and model misspecification. As these elements may confound medication adherence assessments, RUV was intentionally minimized in the present analysis to reduce setting-specific noise and facilitate clearer characterization of covariate effects. To enhance real-world applicability, the accompanying dashboard allows users to adjust the RUV level based on reported values from source population PK studies (Table S2 in Multimedia Appendix 1), known assay variability, or clinical experience.

### Limitations

The study has several limitations. First, there are numerous patterns of nonadherence and we only considered the scenario of missing doses. Other types of nonadherence, such as delayed doses, missed partial doses, and inadvertent overdoses, were not considered. Second, due to the lack of population-PK studies, specifically in pediatric patients, pregnant women, and for ER formulation, we did not include all these scenarios in our analysis. However, our dashboard can be readily extended to include these populations, or novel formulations once their population PK parameters become available. In addition, it is important to note that while we provide accurate estimation of probabilities for recent medication events, the clinical judgment should not be solely based on it. Comprehensive assessment must be performed to incorporate the patient’s overall condition, medication history, and relevant information.

### Future Directions

As epilepsy pharmacotherapy evolves, the dashboard will be updated to incorporate emerging population PK models for novel ASMs when available. Concurrently, it will extend to pharmacodynamic models to bridge the gap between PK and clinical outcomes, thereby quantifying the risks of breakthrough seizures from nonadherence trajectories. Leveraging prior work on remedial dosing regimens on ASMs [18], the tool can be expanded to provide remedial dosing strategies for clinicians to safely restore therapeutic concentrations after missed doses. Finally, the integration with multidimensional data, such as digital biomarkers and electronic health records, will be explored. The comprehensive strategy will ultimately facilitate the realization of precise, patient-specific life-cycle management in epilepsy treatment.

### Conclusion

In conclusion, this study establishes a Bayesian-based PK approach to enhance the objective assessment of ASM adherence. By leveraging TDM data, the approach showed large improvement in assessing nonadherence patterns compared to the previous guidelines. In addition, to bridge the methodology with clinical practice, we developed an interactive dashboard that translates PK principles into visual and interpretable outputs. The work demonstrates the feasibility of transitioning from traditional population-based monitoring to individual-specific management for patients with epilepsy.

## Supplementary material

10.2196/77917 Multimedia Appendix 1 Literature identification, the posterior probabilities-concentration curves, sensitivity analysis results, and the summary of identified population pharmacokinetic models.

## Abbreviations

ASM: antiseizure medications
C_0_: steady-state trough concentration
PK: pharmacokinetic
PPK: population pharmacokinetic
TDM: therapeutic drug monitoring
