# Supplementary material for: Bayesian-Based Pharmacokinetic Framework Integrated with Therapeutic Drug Monitoring for Assessing Adherence to Antiseizure Medications: A Clinical Trial Simulation Study
Source: J Med Internet Res. 2026 Jan 2;28:e77917. doi: 10.2196/77917 (PMC12772940; doi:10.2196/77917)
Supplement: Multimedia Appendix 1 [file jmir-v28-e77917-s001.docx]

**Bayesian-based Pharmacokinetic Framework Integrated with Therapeutic Drug Monitoring for Antiseizure Medications Adherence Assessment in Patients with Epilepsy: Clinical Trial Simulation Study**

Xiao-Qin Liu, PhD^1^; Zi-Ran Li, PhD ^2^; Wei-Wei Lin, PhD ^3^; Juan Wang, BSc^1^; Fu-Qing Gu, MS ^1^; Jun-Jie Ding, PhD ^4^; Zheng Jiao, PhD ^1*^

1. Shanghai Chest Hospital, Shanghai Jiao Tong University School of Medicine, Shanghai, China

2. Department of Bioengineering & Therapeutic Sciences, University of California San Francisco, San Francisco, United States

3. Department of Pharmacy, the First Affiliated Hospital of Fujian Medical University, Fuzhou, China

4. Center for Tropical Disease and Global Health, Nuffield Department of Clinical Medicine, University of Oxford, Oxford UK

**Supplementary files**

[Supplementary Text. The identification of population pharmacokinetic studies. 3](#_Toc215223618)

[Figure S1. PRISMA flow diagram for identifying population pharmacokinetic studies of brivaracetam. 4](#_Toc215223619)

[Figure S2. PRISMA flow diagram for identifying population pharmacokinetic studies of lacosamide. 5](#_Toc215223620)

[Figure S3. PRISMA flow diagram for identifying population pharmacokinetic studies of lamotrigine. 6](#_Toc215223621)

[Figure S4. PRISMA flow diagram for identifying population pharmacokinetic studies of levetiracetam. 7](#_Toc215223622)

[Figure S5. PRISMA flow diagram for identifying population pharmacokinetic studies of oxcarbazepine. 8](#_Toc215223623)

[Figure S6. PRISMA flow diagram for identifying population pharmacokinetic studies of perampanel. 9](#_Toc215223624)

[Figure S7. PRISMA flow diagram for identifying population pharmacokinetic studies of topiramate. 10](#_Toc215223625)

[Figure S8. PRISMA flow diagram for identifying population pharmacokinetic studies of valproic acid. 11](#_Toc215223626)

[Figure S9. PRISMA flow diagram for identifying population pharmacokinetic studies of vigabatrin. 12](#_Toc215223627)

[Figure S10. The posterior probabilities-concentration curves of brivaracetam. 13](#_Toc215223628)

[Figure S11. The posterior probabilities-concentration curves of carbamazepine. 14](#_Toc215223629)

[Figure S12. The posterior probabilities-concentration curves of clobazam. 15](#_Toc215223630)

[Figure S13. The posterior probabilities-concentration curves of eslicarbazepine acetate. 16](#_Toc215223631)

[Figure S14. The posterior probabilities-concentration curves of lacosamide. 17](#_Toc215223632)

[Figure S15. The posterior probabilities-concentration curves of lamotrigine. 18](#_Toc215223633)

[Figure S16. The posterior probabilities-concentration curves of levetiracetam. 19](#_Toc215223634)

[Figure S17. The posterior probabilities-concentration curves of oxcarbazepine. 20](#_Toc215223635)

[Figure S18. The posterior probabilities-concentration curves of perampanel. 21](#_Toc215223636)

[Figure S19. The posterior probabilities-concentration curves of phenobarbital. 22](#_Toc215223637)

[Figure S20. The posterior probabilities-concentration curves of topiramate. 23](#_Toc215223638)

[Figure S21. The posterior probabilities-concentration curves of valproic acid. 24](#_Toc215223639)

[Figure S22. The posterior probabilities-concentration curves of vigabatrin. 25](#_Toc215223640)

[Figure S23. The posterior probabilities-concentration curves of zonisamide. 26](#_Toc215223641)

[Figure S24. Effect of renal function on the distinguishability of different dosing scenarios. 27](#_Toc215223642)

[Figure S25. Effect of concomitant medicine on the distinguishability of different dosing scenarios. 28](#_Toc215223643)

[Figure S26. Effect of sampling time on the distinguishability of dosing behaviors. 29](#_Toc215223644)

[Figure S27. Effect of prior probability on the distinguishability of dosing behaviors. 30](#_Toc215223645)

[Figure S28. The effect of prior probability on posterior probability in case 1. 31](#_Toc215223646)

[Figure S29. The effect of residual explained variability on posterior probability in case 1. 32](#_Toc215223647)

[Figure S30. The effect of prior probability on posterior probability in case 2. 33](#_Toc215223648)

[Figure S31. The effect of residual explained variability on posterior probability in case 2. 34](#_Toc215223649)

[Table S1. The identified population pharmacokinetic studies of antiseizure medications and corresponding dosing regimens. 35](#_Toc215223650)

[Table S2. Population pharmacokinetic parameter estimates of the identified ASMs studies. 36](#_Toc215223651)

[Reference 42](#_Toc215223652)

# Supplementary Text. The identification of population pharmacokinetic studies.

The population pharmacokinetic (PPK) models of commonly used antiseizure medications (ASMs) were indentified in our previous study[1], including carbamazepine, lamotrigine, eslicarbazepine acetate, levetiracetam, oxcarbazepine, phenobarbital, topiramate, valproic acid, clobazam, and zonisamide.

Therefore, updated literature search was performed for these ASMs from 31 March, 2022 to 30 November, 2024. The population PK studies were identified using the following search terms: “drug name” and ‘population pharmacokinetic’, ‘pharmacokinetic modeling’, ‘nonlinear mixed effect model’, ‘NONMEM’, ‘WINNONMIX’, ‘P-PHARM’, ‘nlmixed’, ‘NLME’, ‘USC*PACK’ or ‘MONOLIX’. Furthermore, the reference lists of the selected articles were reviewed to uncover any pertinent studies. The literature search was conducted independently by two authors. In cases of disagreement, a third senior investigator was consulted for resolution. The updated literature was selected if they enrolled a larger number of participants compared with the previously identified PPK studies, or based on the judgement from the researchers.

The PPK models of 4 additional ASMs were not identified in previous study, including brivaracetam, lacosamide, perampanel and vigabatrin. Therefore, the PPK studies of these ASMs were screened in the PubMed and Embase from database inception until 30 November, 2024.

After screening, no newly published population PK models were identified for carbamazepine, clobazam, eslicarbazepine acetate, phenobarbital and zonisamide.

A study was considered eligible for inclusion if it met the following criteria: (1) the
study population included patients with epilepsy; (2) the study was focused on
population PK or pharmacokinetic/pharmacodynamic (PK/PD) analysis; (3) the study
enrolled more than 30 participates; (4) the concentration-time profiles of typical
patients based on the established model showed no obvious deviation from the others.

A study was excluded if: (1) it was a review or only focused on the methodology,
algorithm, or software considerations; (2) it was published in a non-English language;
(3) the information on methodology or pharmacokinetics was insufficient; (4) only
injection formulation was used; and (5) only neonates were enrolled.

When several studies were identified for one ASM, preference was given to studies
that prospectively investigated in multicenter or studies that included intensive
sampling. This process was also performed by two independent reviewers. Moreover,
a third senior investigator was consulted to resolve the discrepancies.

# Figure S1. PRISMA flow diagram for identifying population pharmacokinetic studies of brivaracetam.


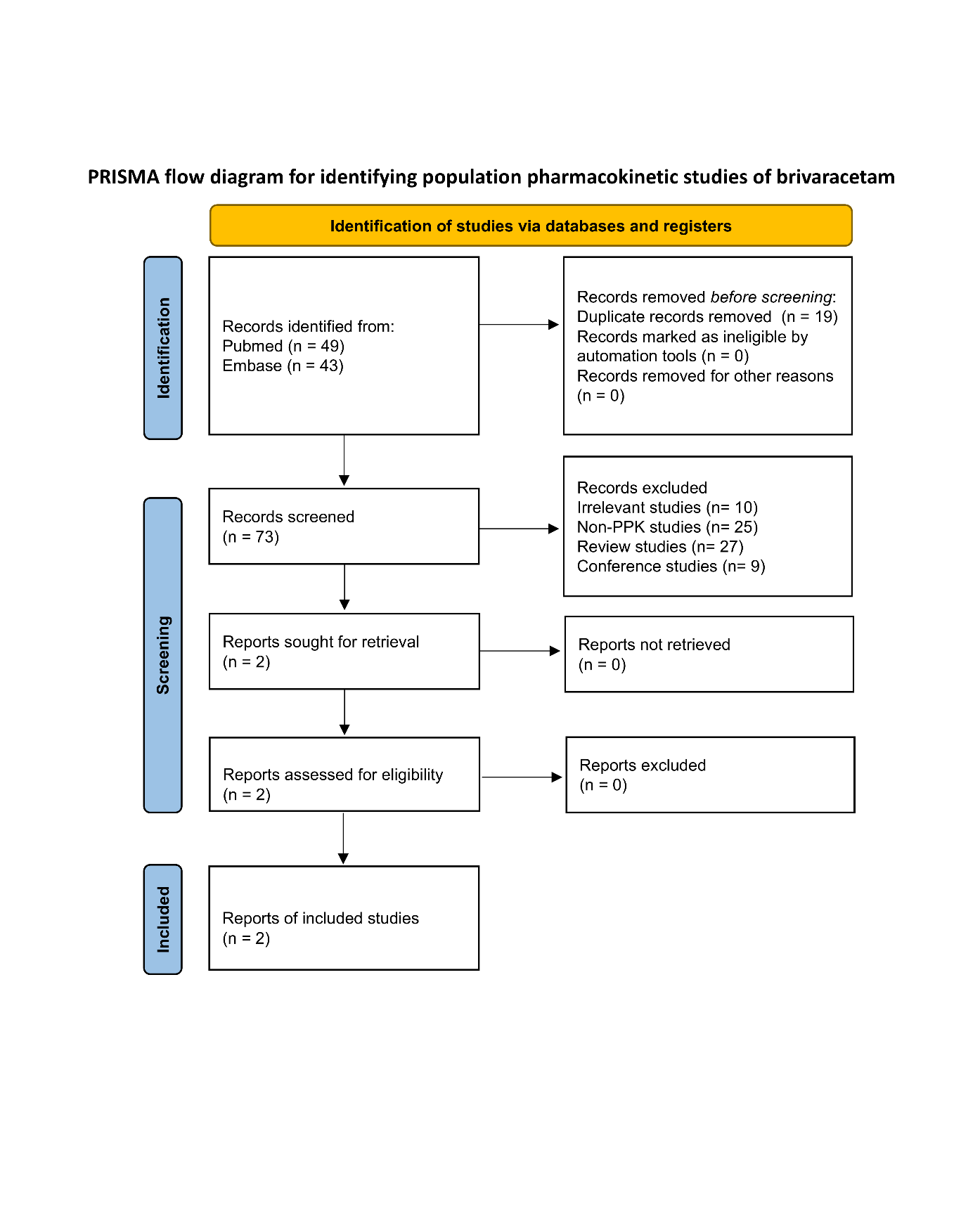


# Figure S2. PRISMA flow diagram for identifying population pharmacokinetic studies of lacosamide.


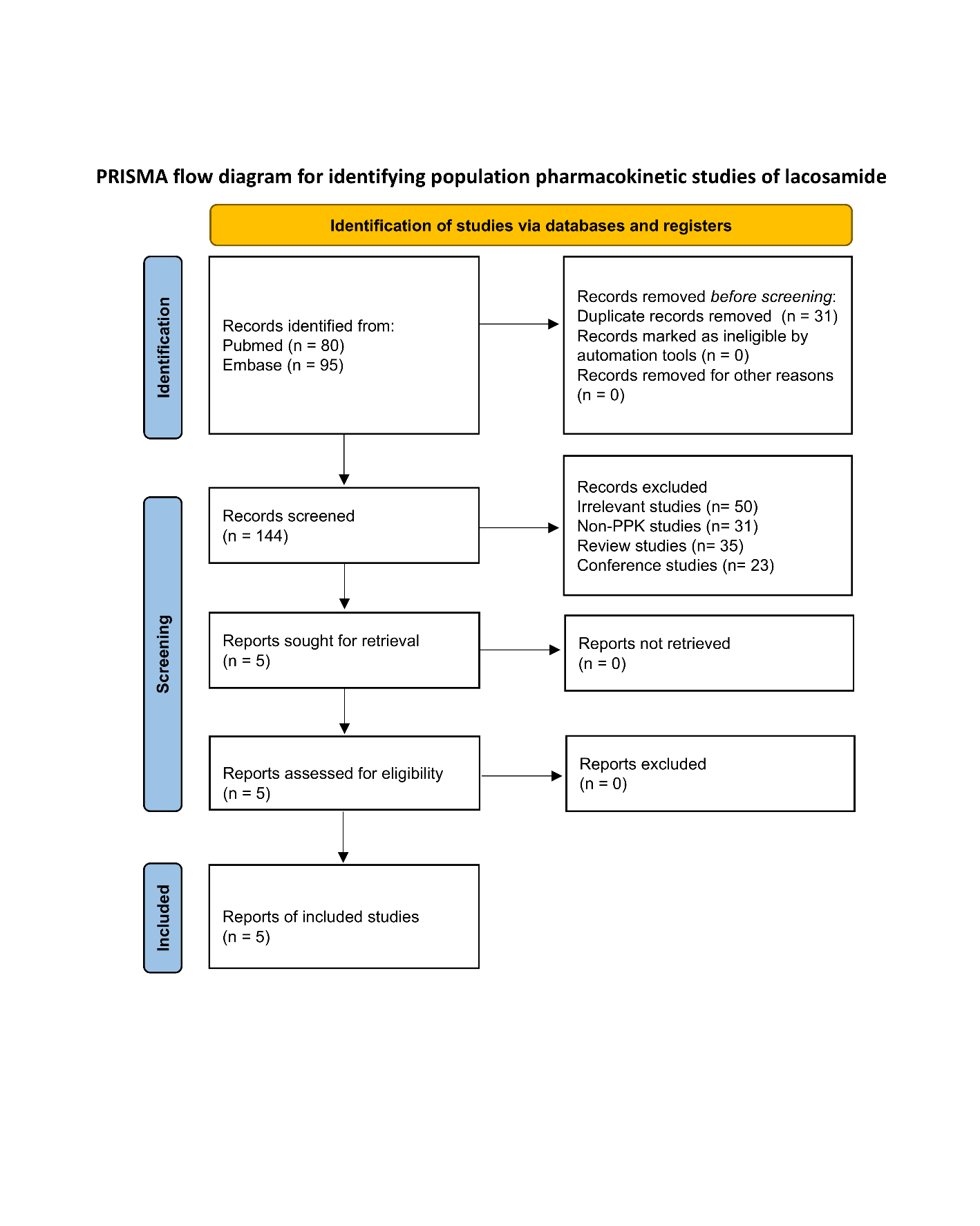


# Figure S3. PRISMA flow diagram for identifying population pharmacokinetic studies of lamotrigine.


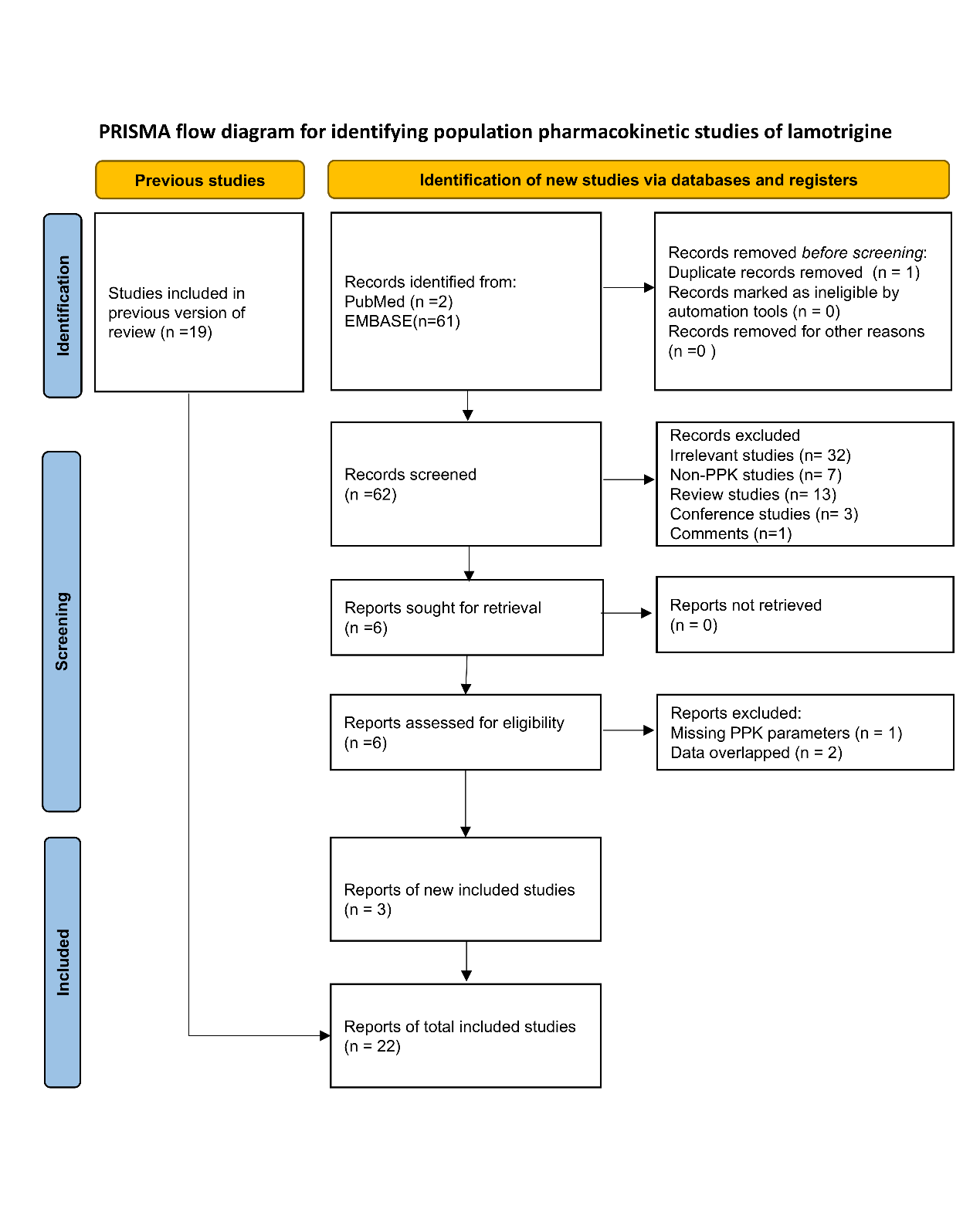


# Figure S4. PRISMA flow diagram for identifying population pharmacokinetic studies of levetiracetam.


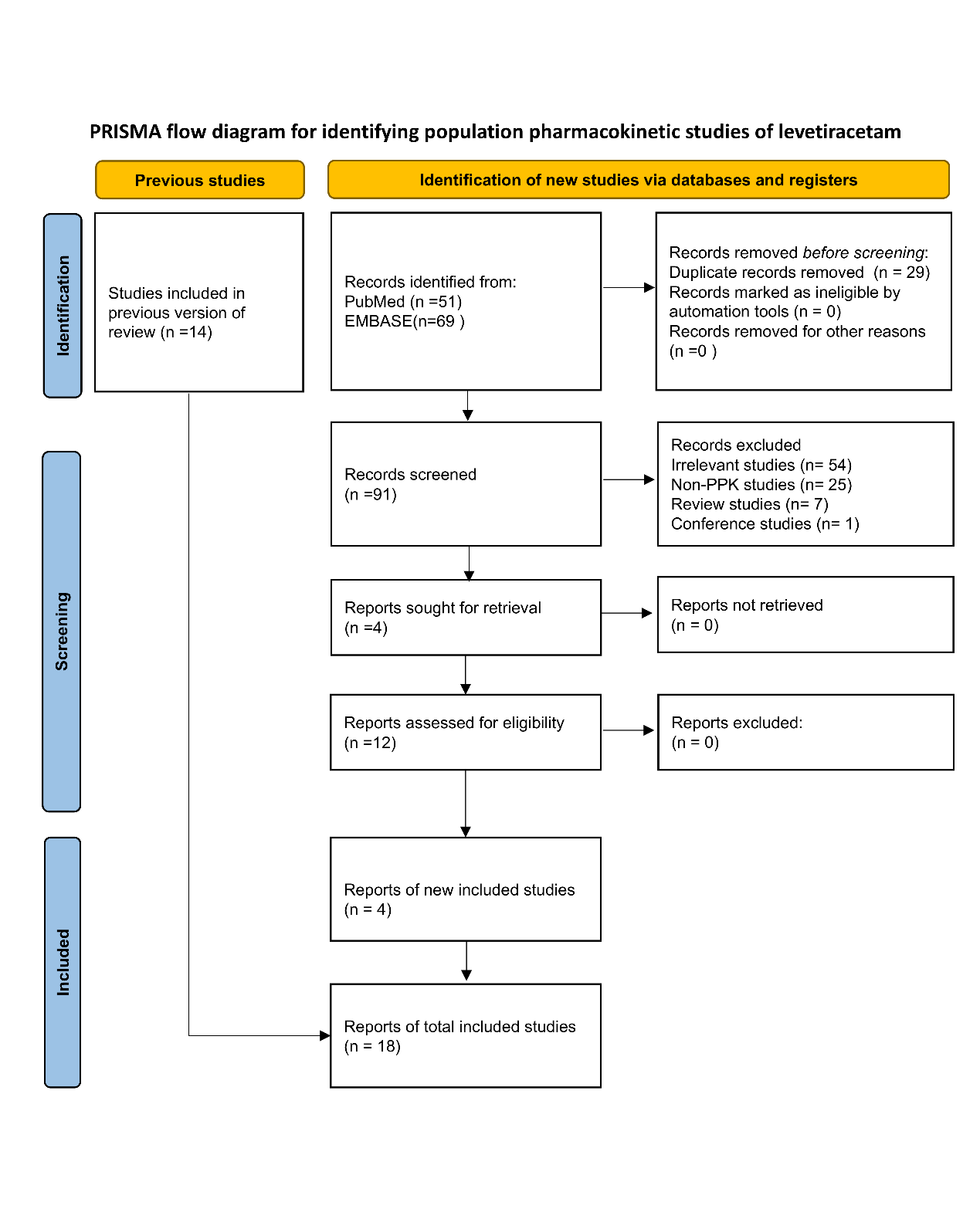


# Figure S5. PRISMA flow diagram for identifying population pharmacokinetic studies of oxcarbazepine.


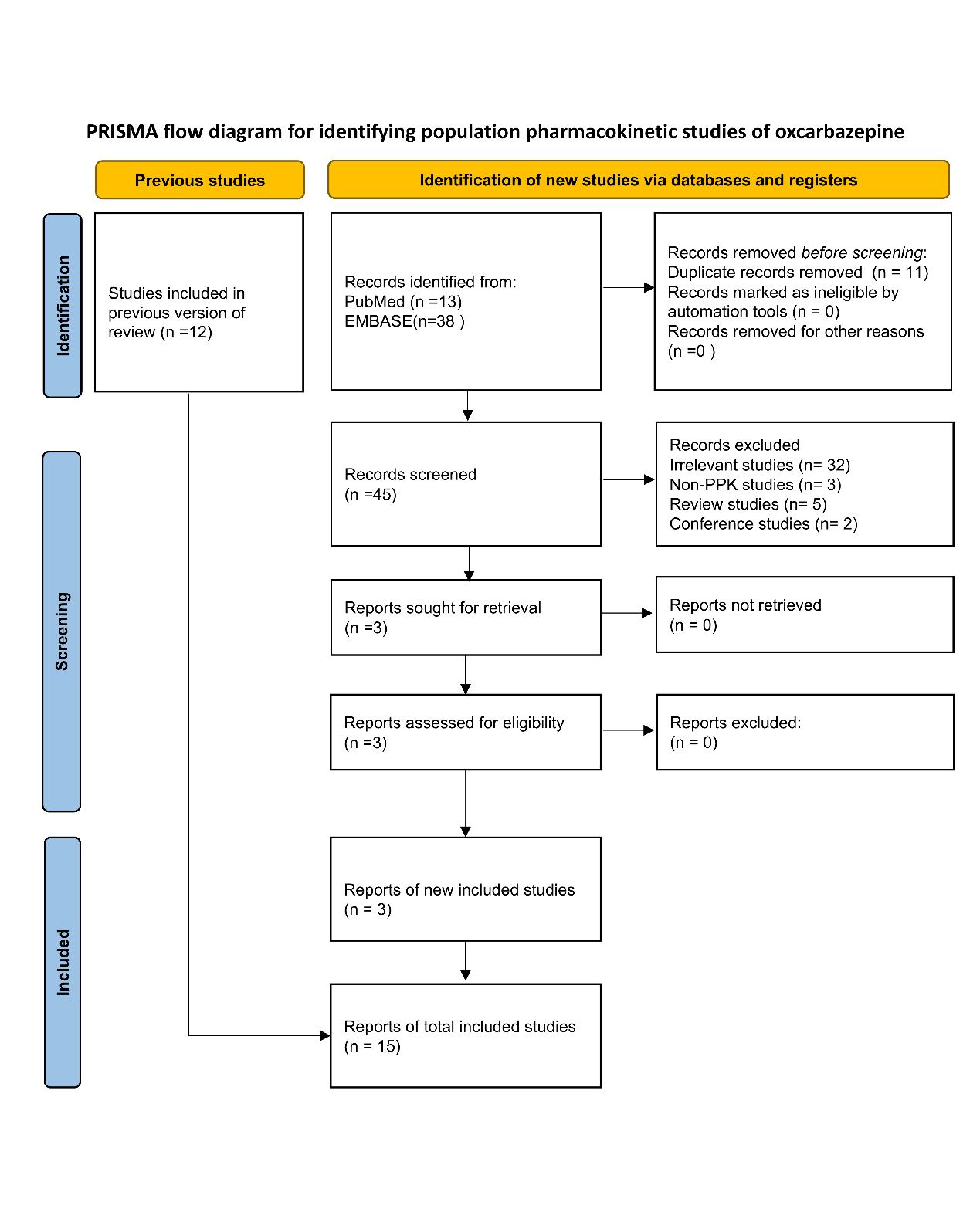


# Figure S6. PRISMA flow diagram for identifying population pharmacokinetic studies of perampanel.


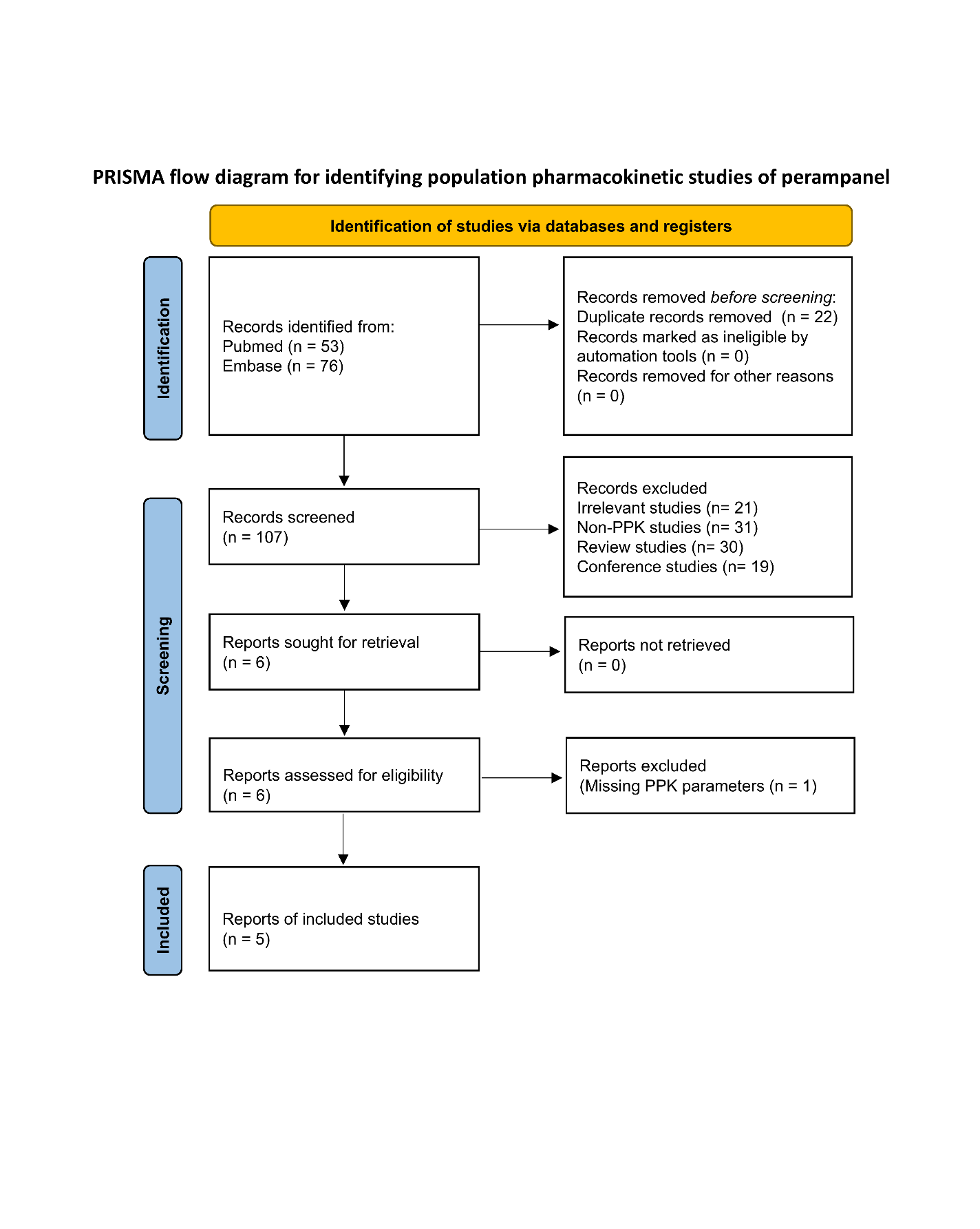


# Figure S7. PRISMA flow diagram for identifying population pharmacokinetic studies of topiramate.


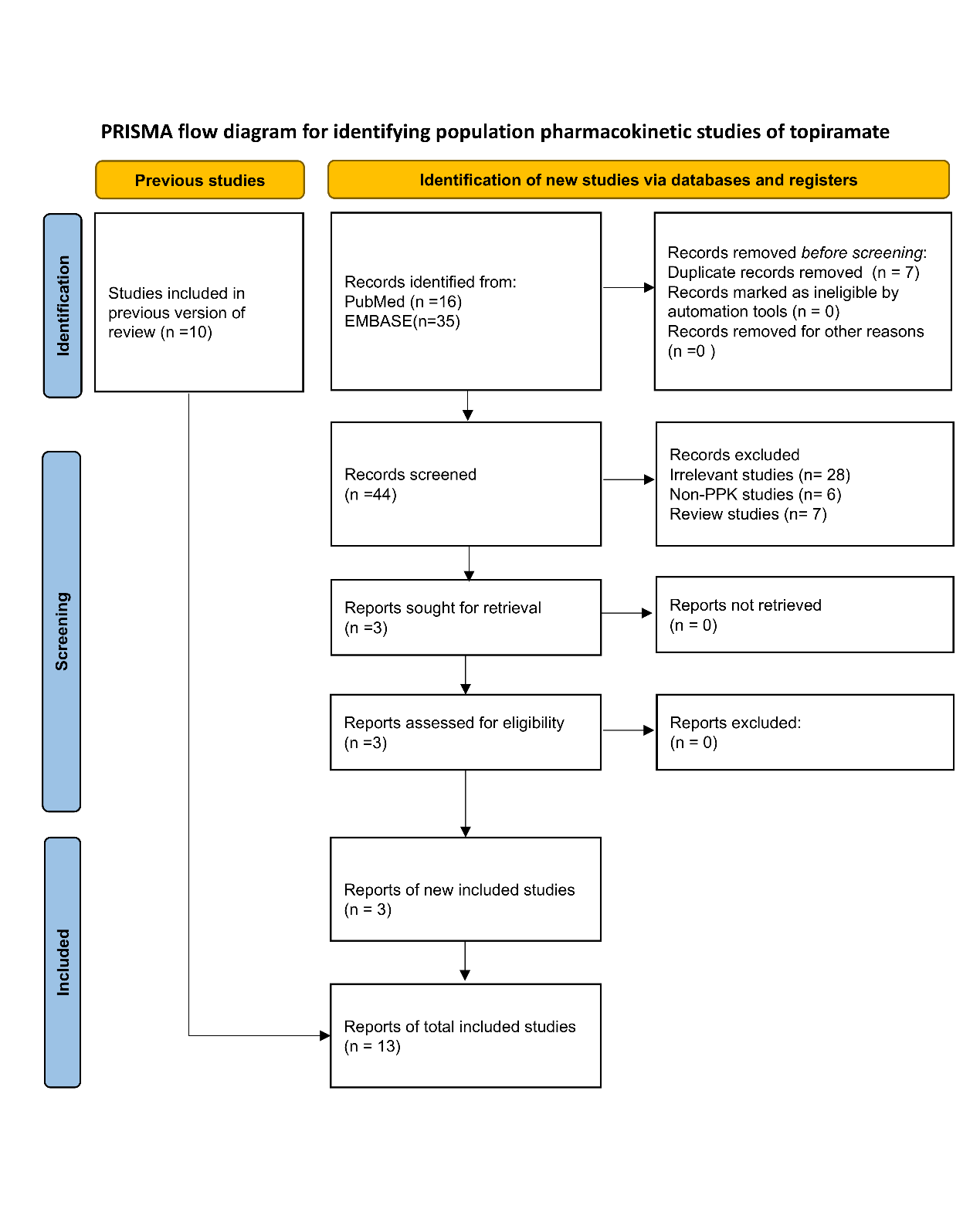


# Figure S8. PRISMA flow diagram for identifying population pharmacokinetic studies of valproic acid.


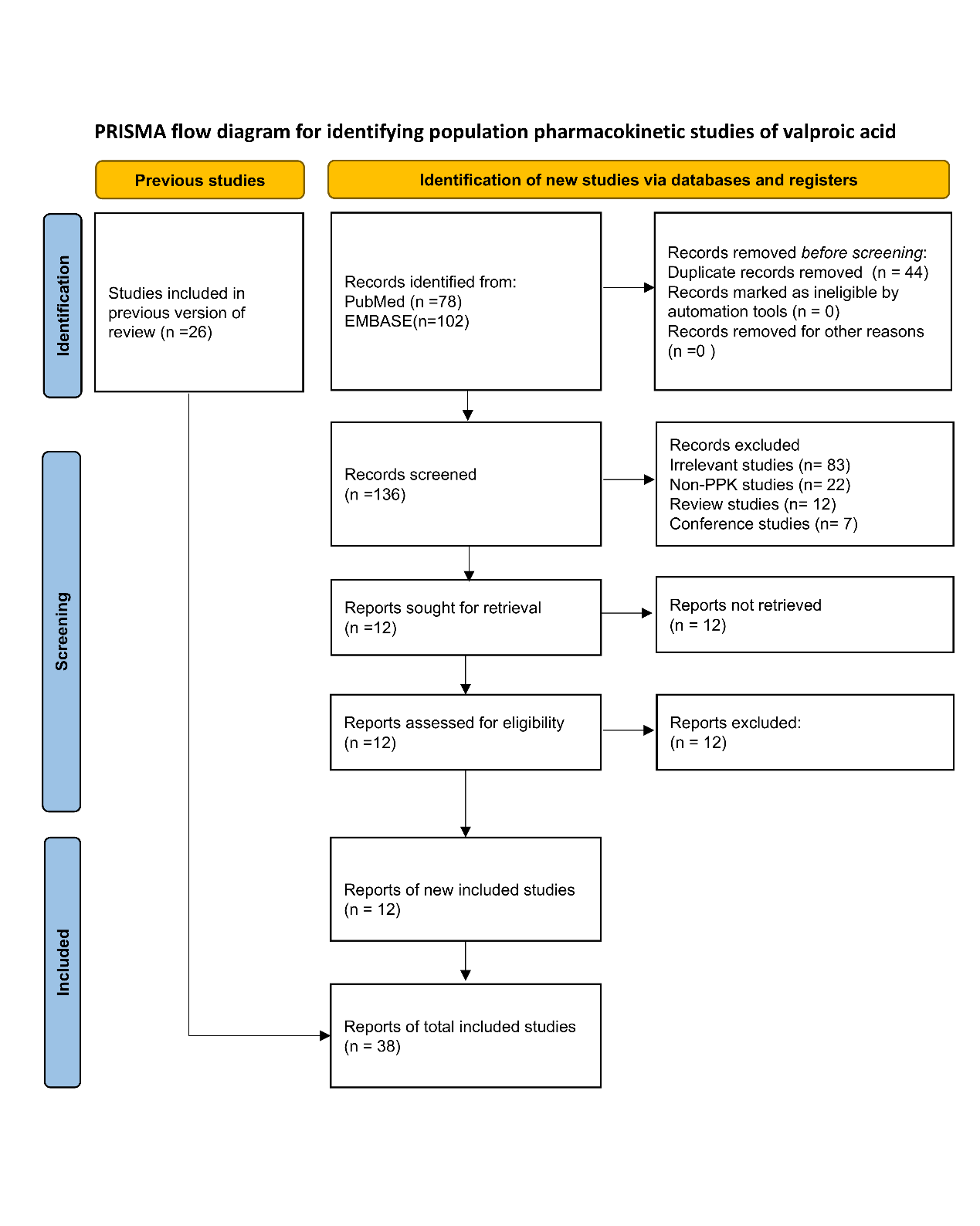


# Figure S9. PRISMA flow diagram for identifying population pharmacokinetic studies of vigabatrin.


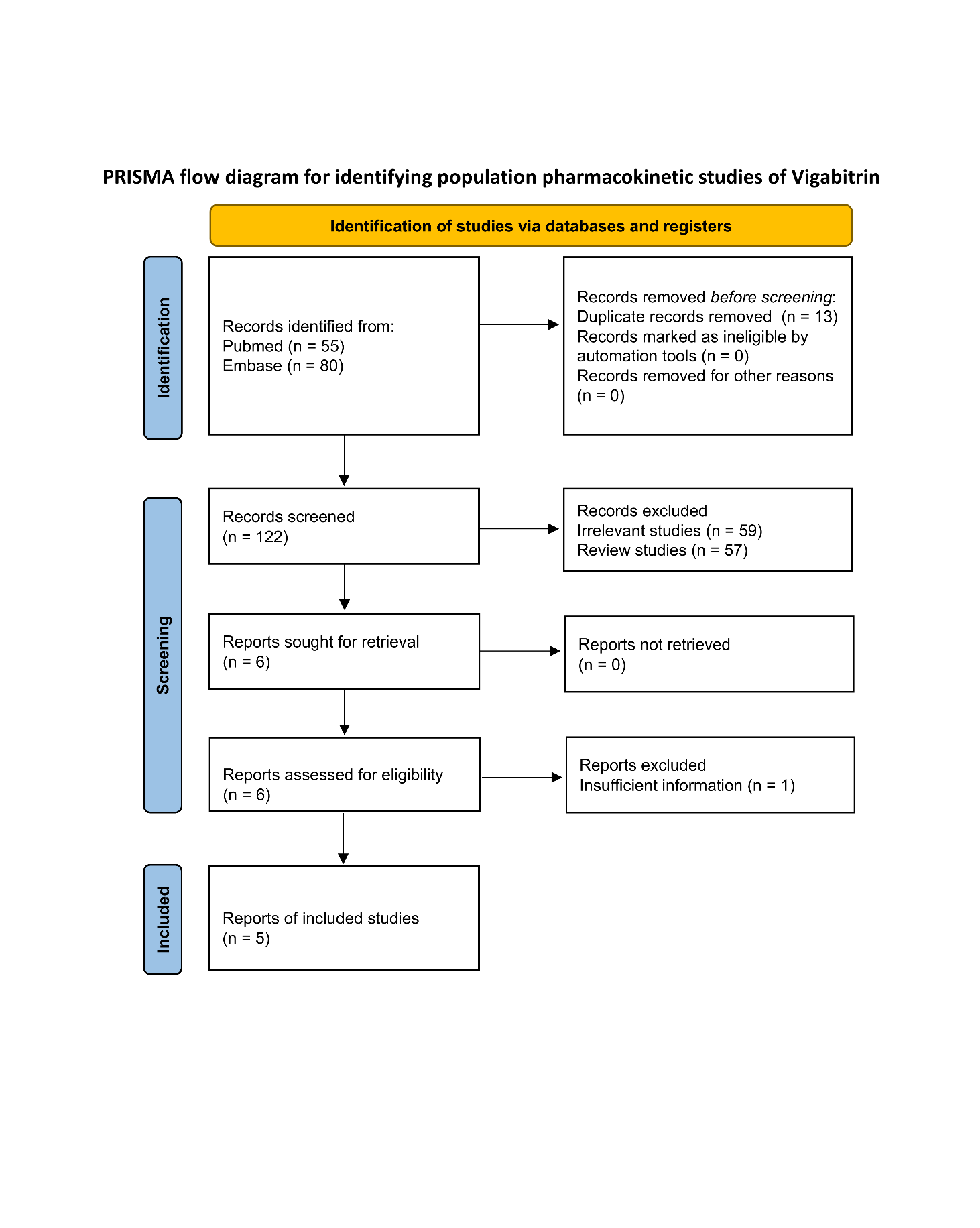


# Figure S10. The posterior probabilities-concentration curves of brivaracetam.


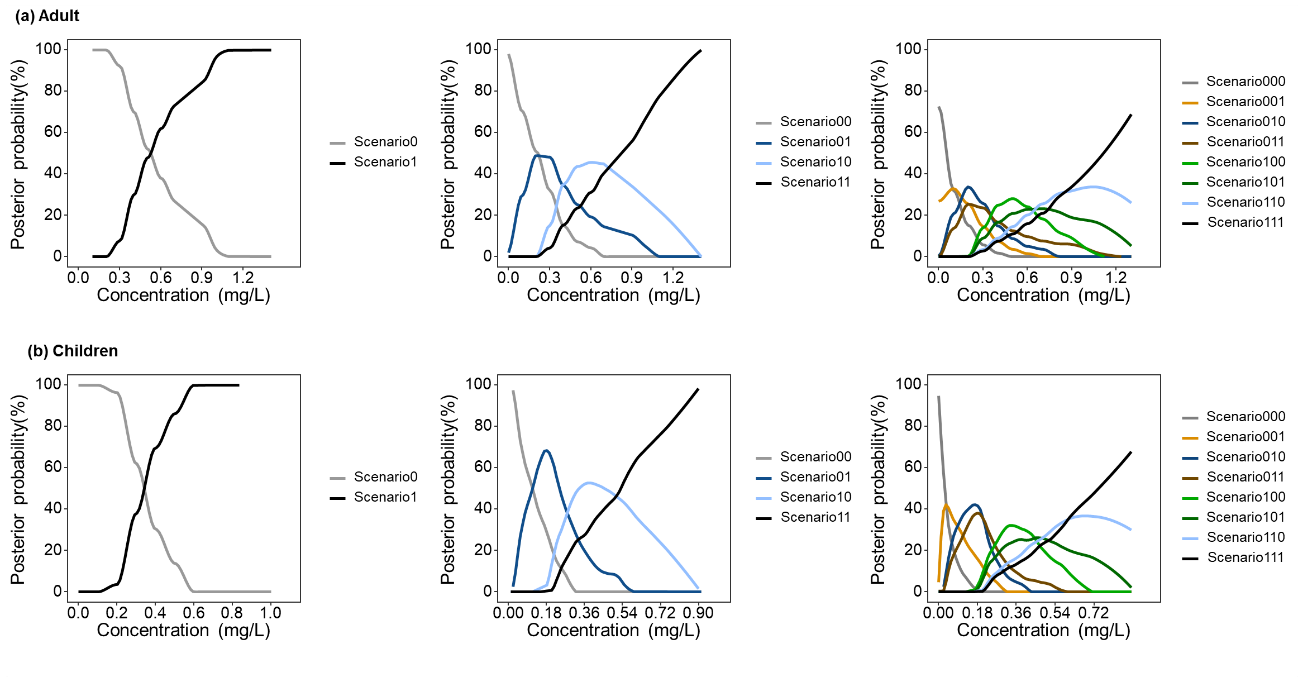
(a) 40 y, weighing 70 kg, measuring 180 cm, and taking brivaracetam 50 mg q12h; (b) 8 y, weighing 25 kg, measuring 127cm, and taking brivaracetam 25 mg q12h.

# Figure S11. The posterior probabilities-concentration curves of carbamazepine.


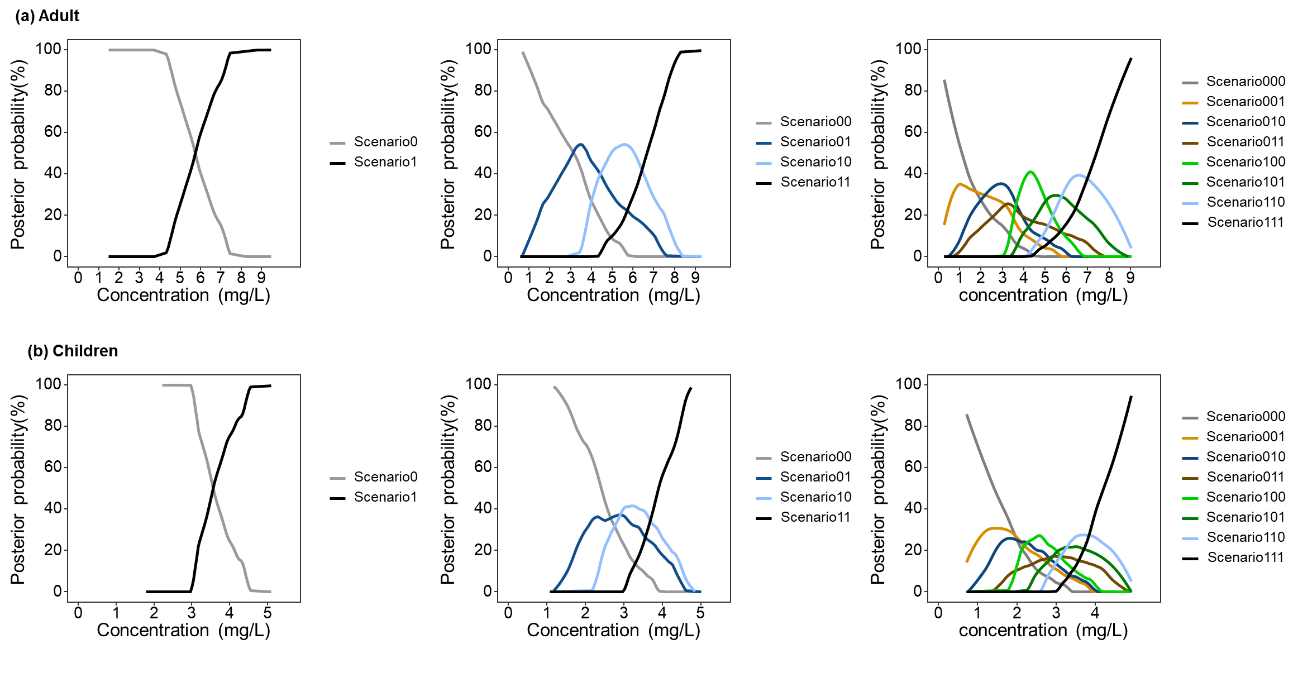


(a) 40 y, weighing 70 kg, measuring 180 cm, and taking carbamazepine 400 mg q12h; (b) 8 y, weighing 25 kg, measuring 127cm, and taking carbamazepine 100 mg q12h.

# Figure S12. The posterior probabilities-concentration curves of clobazam.


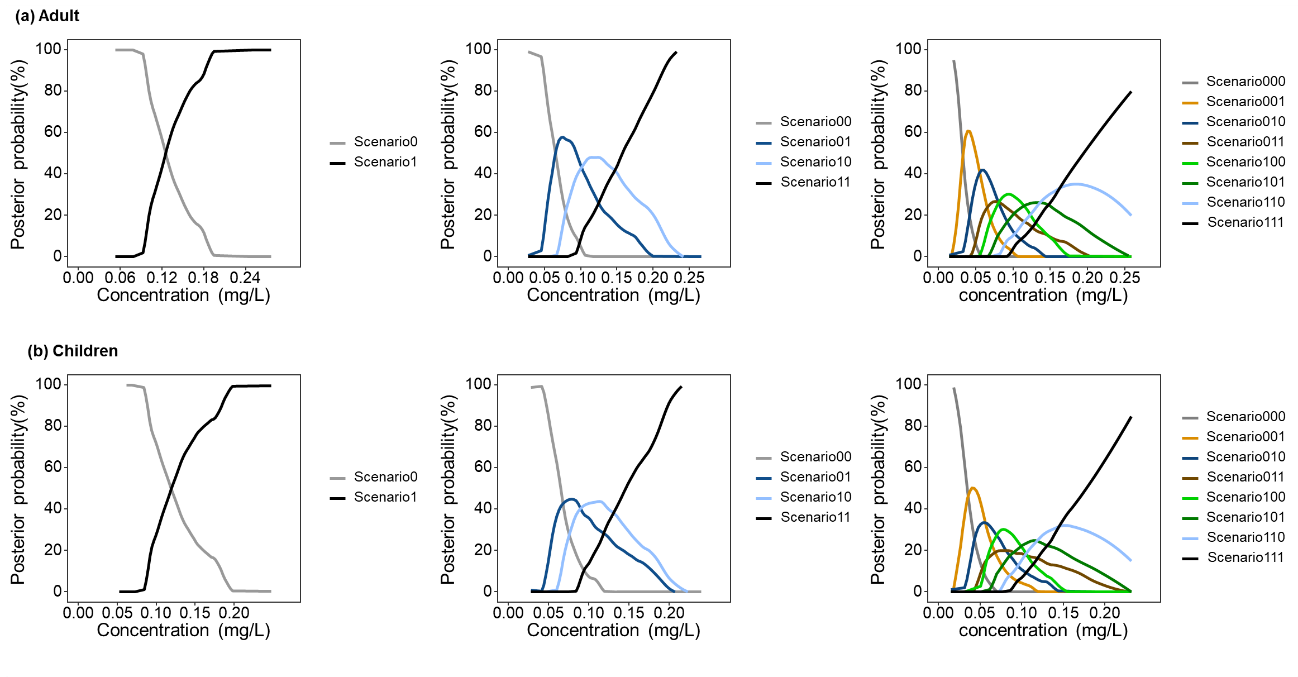


(a) 40 y, weighing 70 kg, measuring 180 cm, and taking clobazam 10 mg q12h; (b) 8 y, weighing 25 kg, measuring 127cm, and taking clobazam 5 mg q12h.

# Figure S13. The posterior probabilities-concentration curves of eslicarbazepine acetate.


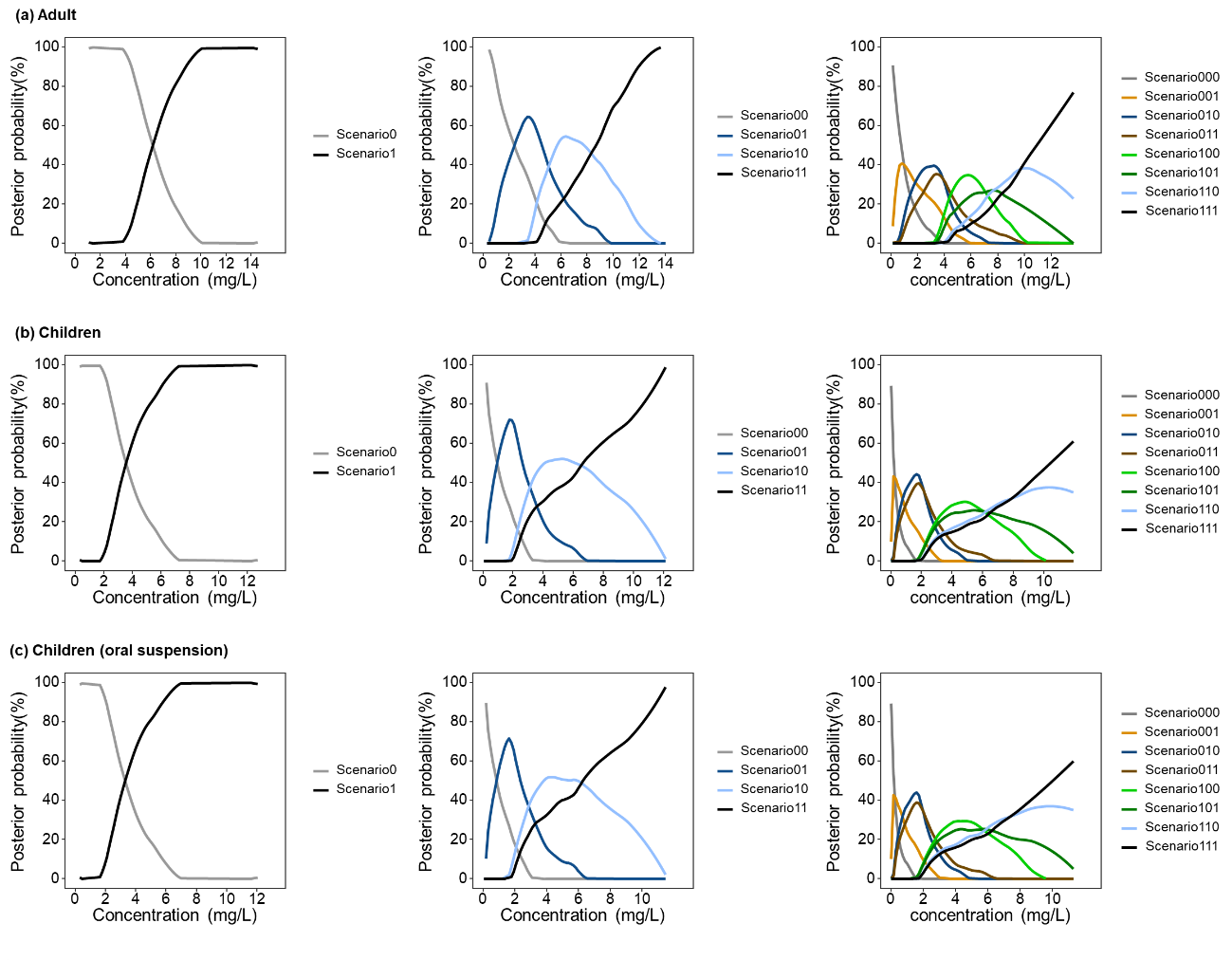


(a) 40 y, weighing 70 kg, measuring 180 cm, and taking eslicarbazepine acetate tablet 800 mg q24h; (b) 8 y, weighing 25 kg, measuring 127cm, and taking eslicarbazepine acetate tablet 400 mg q24h; (c) 8 y, weighing 25 kg, measuring 127cm, and taking eslicarbazepine acetate oral suspension 400 mg q24h.

# Figure S14. The posterior probabilities-concentration curves of lacosamide.


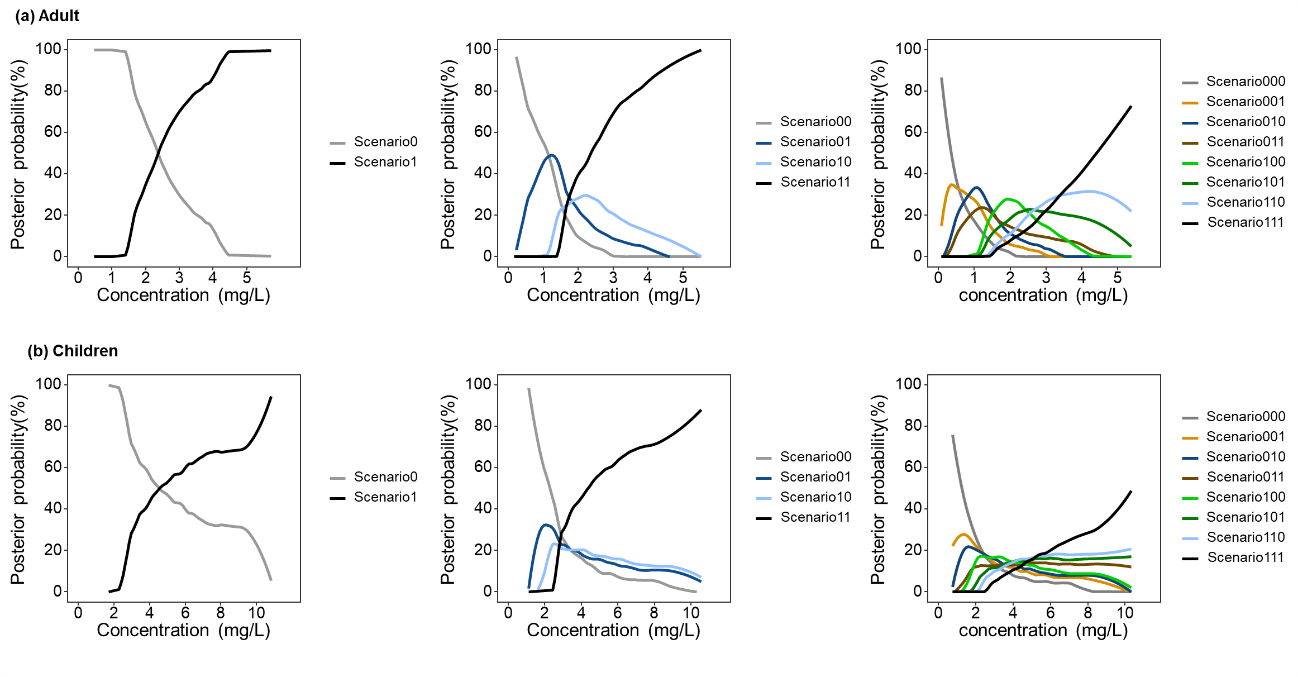
 (a) 40 y, weighing 70 kg, measuring 180 cm, and taking lacosamide 150 mg q12h; (b) 8 y, weighing 25 kg, measuring 127 cm, and taking lacosamide 100 mg q12h.

# Figure S15. The posterior probabilities-concentration curves of lamotrigine.


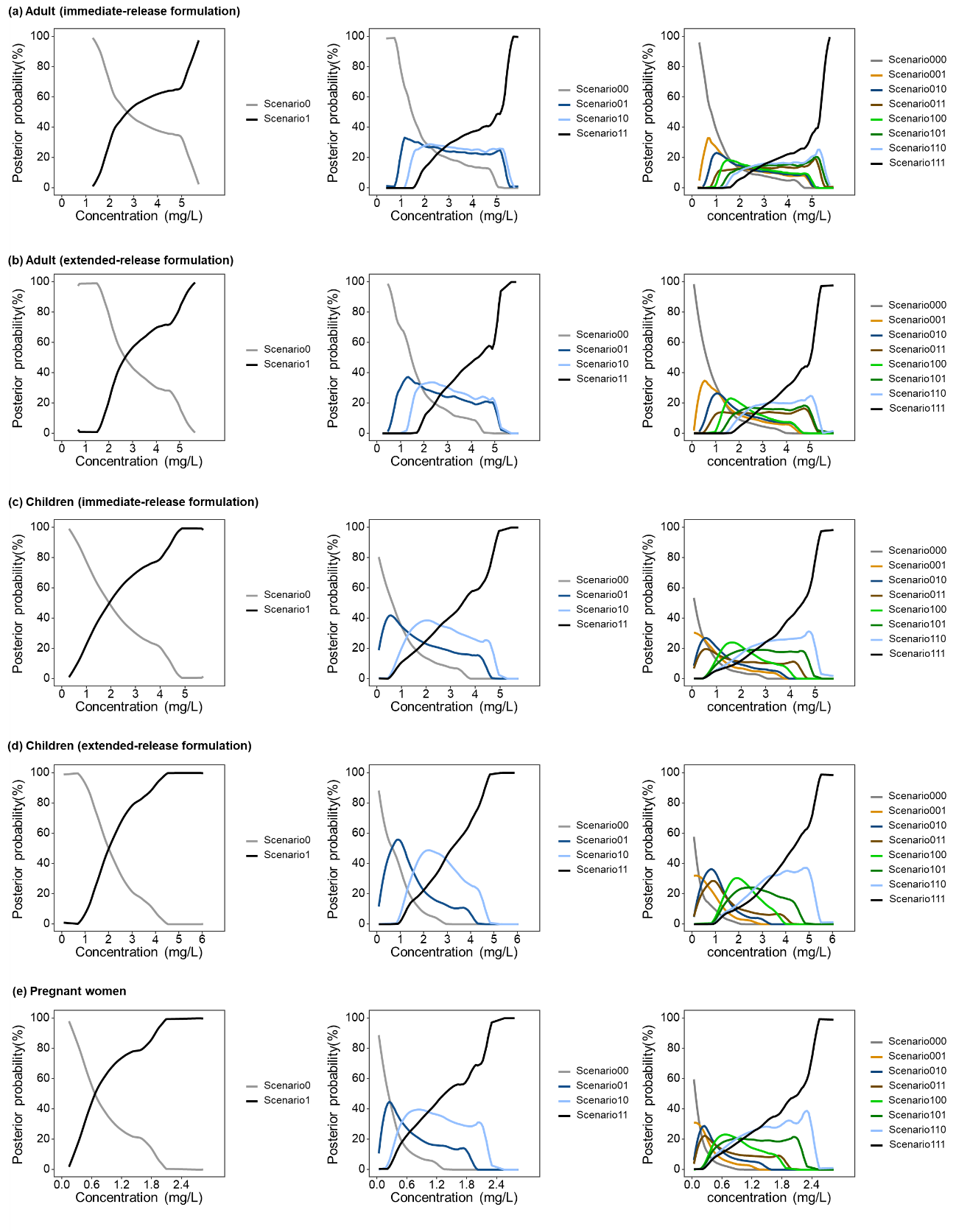


(a) 40 y, weighing 70 kg, measuring 180 cm, and taking lamotrigine (immediate-release formulation) 100 mg q12h; (b) 40 y, weighing 70 kg, measuring 180 cm, and taking lamotrigine (extended-release formulation) 200 mg q24h; (c) 8 y, weighing 25 kg, measuring 127cm, and taking lamotrigine (immediate-release formulation) 50 mg q12h; (d) 8 y, weighing 25 kg, measuring 127cm, and taking lamotrigine (extended-release formulation) 50 mg q12h; (e) pregnant women, aged 25 y, weighing 70 kg, measuring 160 cm, being 30 weeks pregnant, and taking lamotrigine 100 mg q12h.

# Figure S16. The posterior probabilities-concentration curves of levetiracetam.


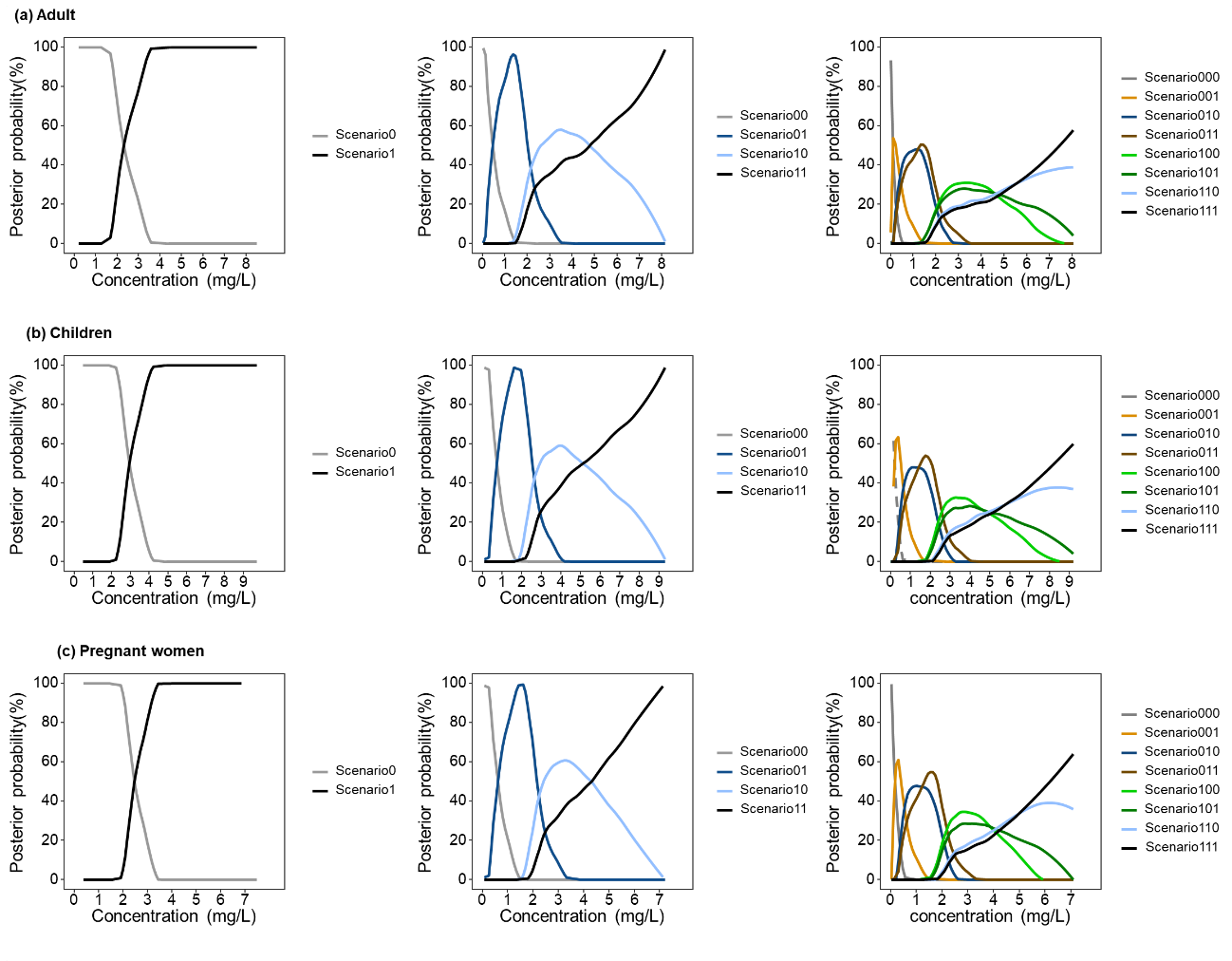


(a) 40 y, weighing 70 kg, measuring 180 cm, and taking levetiracetam 500 mg q12h; (b) 8 y, weighing 25 kg, measuring 127cm, and taking levetiracetam 250 mg q12h; (c) pregnant women, aged 25 y, weighing 70 kg, measuring 160 cm, being 30 weeks pregnant, and taking levetiracetam 500 mg q12h.

# Figure S17. The posterior probabilities-concentration curves of oxcarbazepine.

**
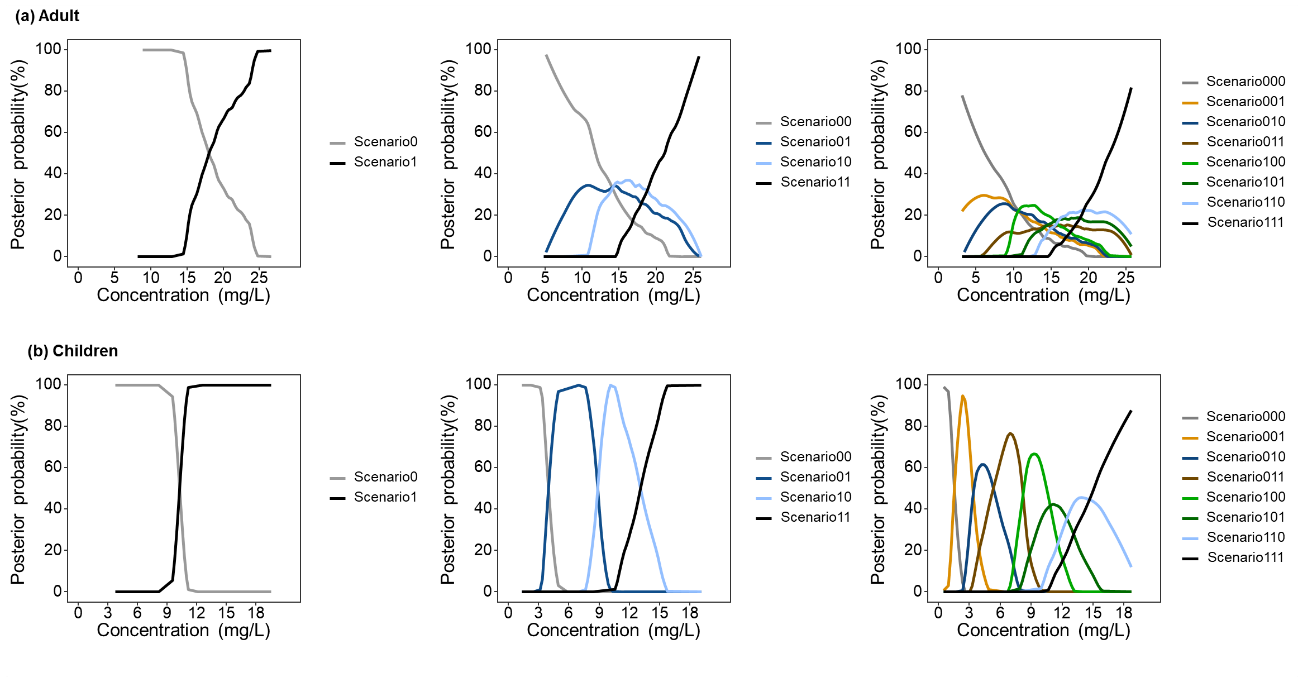
**

(a) 40 y, weighing 70 kg, measuring 180 cm, and taking oxcarbazepine 600 mg q12h; (b) 8 y, weighing 25 kg, measuring 127 cm, and taking oxcarbazepine 300 mg q12h.

# Figure S18. The posterior probabilities-concentration curves of perampanel.


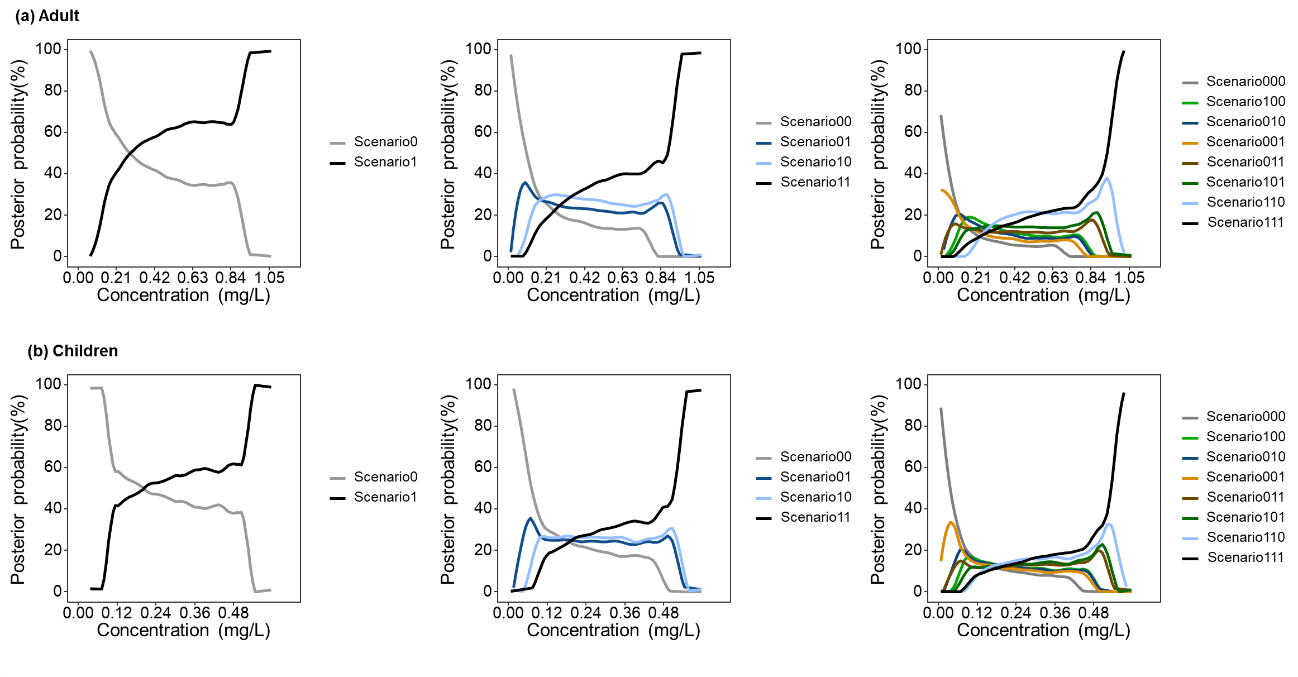


(a) 40 y, weighing 70 kg, measuring 180 cm, and taking perampanel 8 mg q24h; (b) 8 y, weighing 25 kg, measuring 127 cm, and taking perampanel 4 mg q24h.

# Figure S19. The posterior probabilities-concentration curves of phenobarbital.


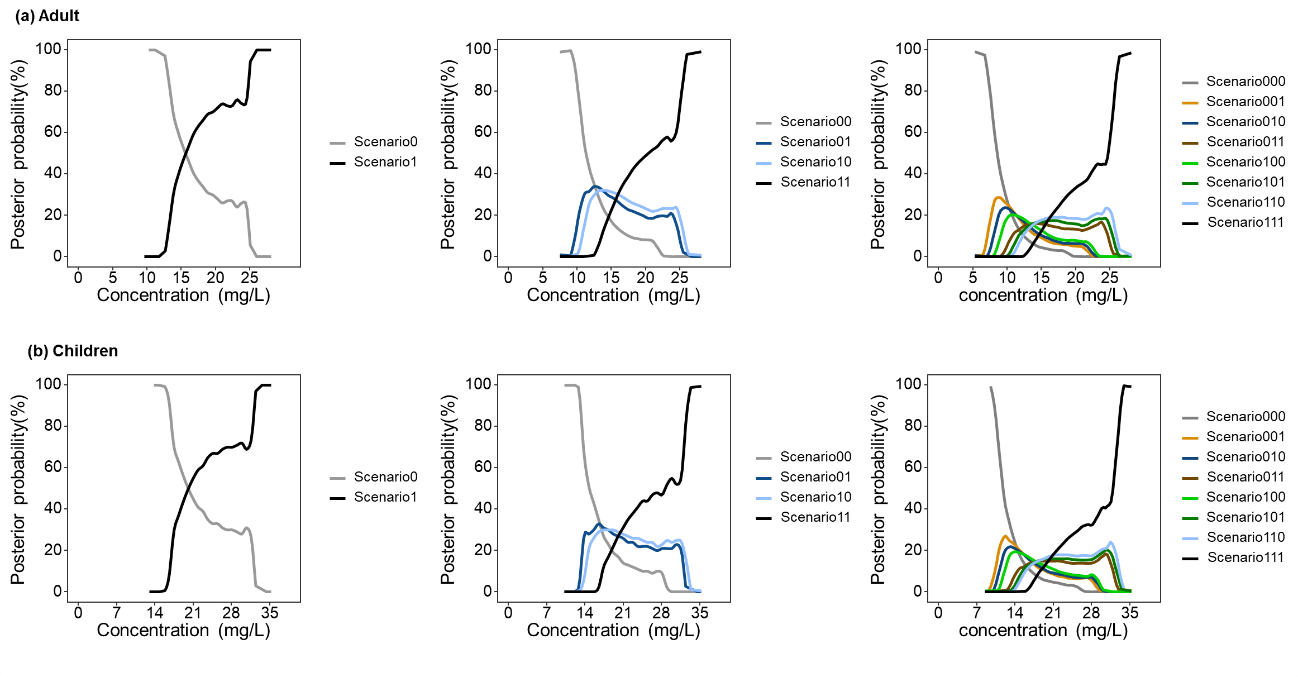


(a) 40 y, weighing 70 kg, measuring 180 cm, and taking phenobarbital 60 mg q12h; (b) 8 y, weighing 25 kg, measuring 127 cm, and taking phenobarbital 60 mg q12h.

# Figure S20. The posterior probabilities-concentration curves of topiramate.


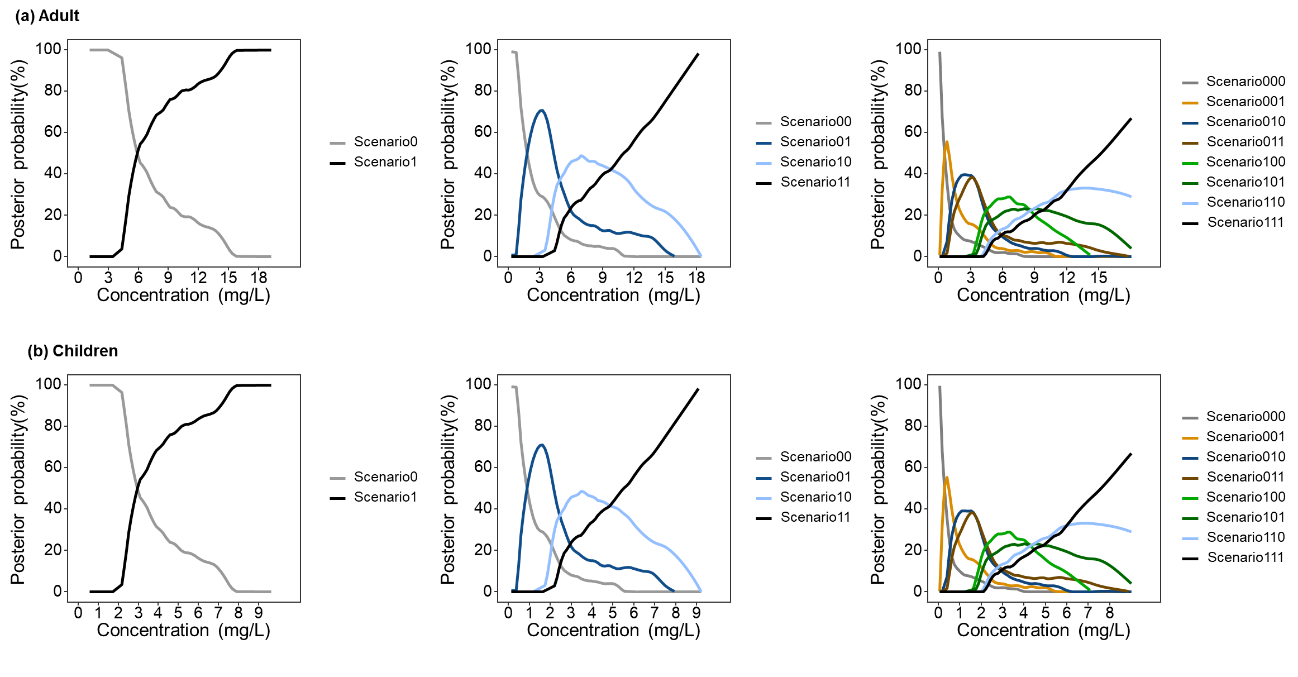


(a) 40 y, weighing 70 kg, measuring 180 cm, and taking topiramate 200 mg q12h; (b) 8 y, weighing 25 kg, measuring 127 cm, and taking topiramate 100 mg q12h.

# Figure S21. The posterior probabilities-concentration curves of valproic acid.


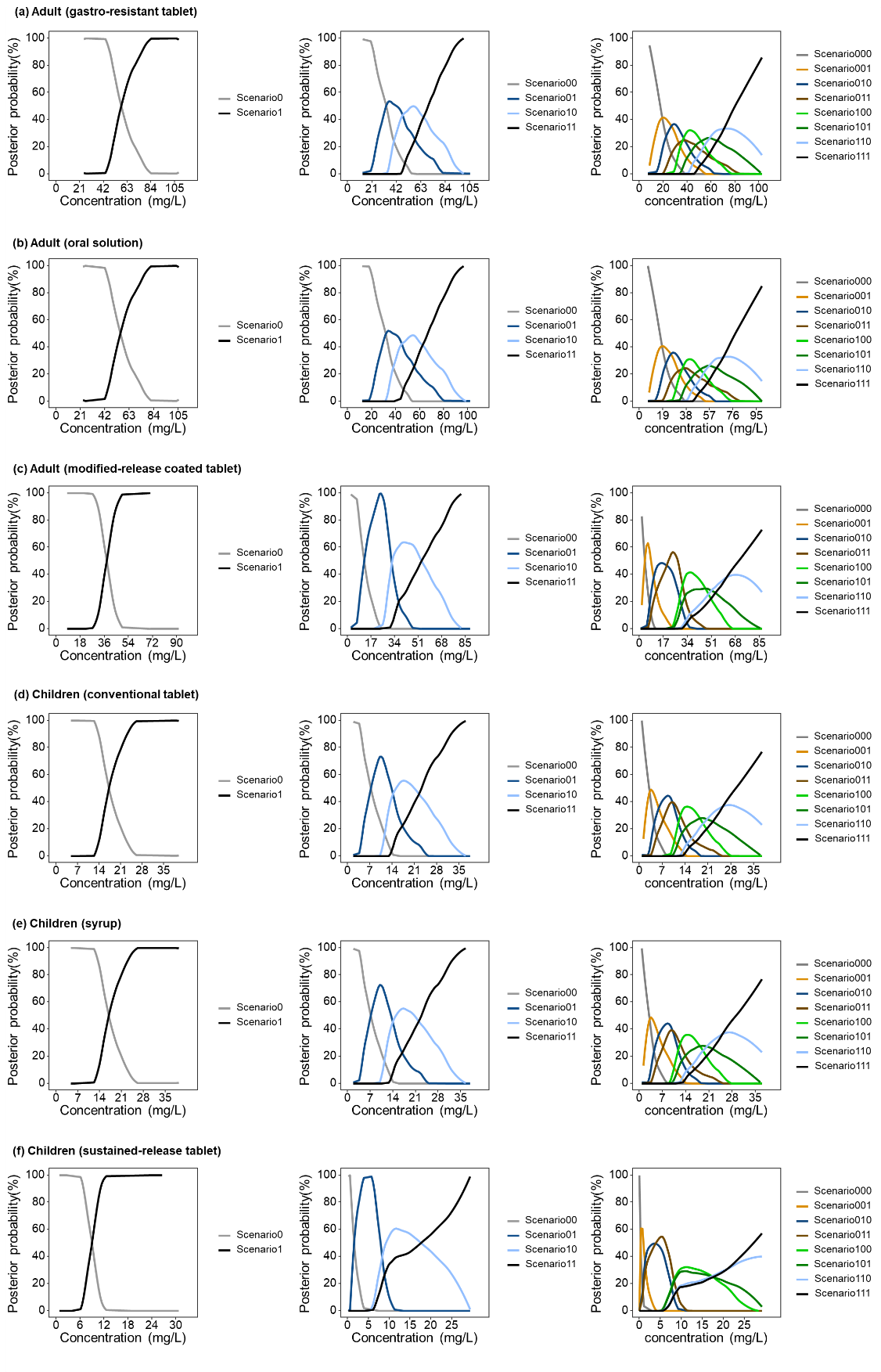


(a) 40 y, weighing 70 kg, measuring 180 cm, and taking valproic acid gastro-resistant tablet 500 mg q12h; (b) 40 y, weighing 70 kg, measuring 180 cm, and taking valproic acid oral solution 500 mg q12h; (c) 40 y, weighing 70 kg, measuring 180 cm, and taking valproic acid modified-release coated tablet 500 mg q12h; (d) 8 y, weighing 25 kg, measuring 127 cm, and taking valproic acid conventional tablet 250 mg q12h; (e) 8 y, weighing 25 kg, measuring 127 cm, and taking valproic acid syrup 250 mg q12h; (f) 8 y, weighing 25 kg, measuring 127 cm, and taking valproic acid sustained-release tablet 500mg q24h.

# Figure S22. The posterior probabilities-concentration curves of vigabatrin.


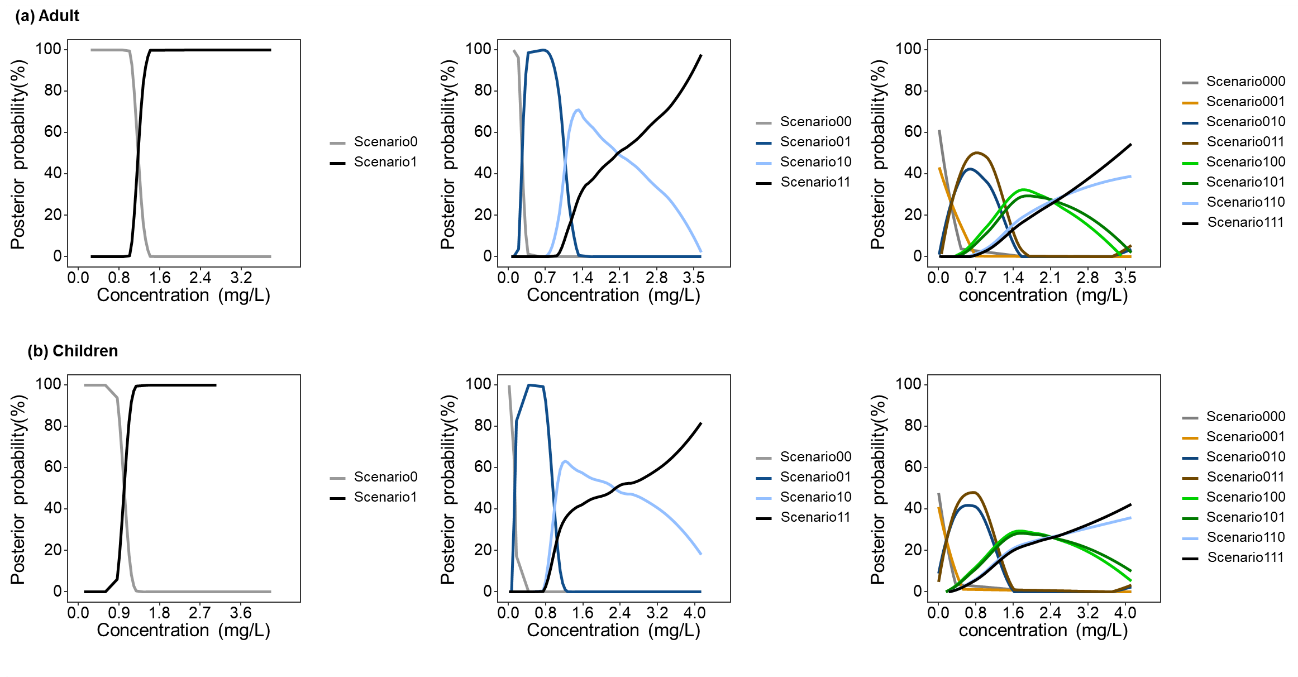


(a) 40 y, weighing 70 kg, measuring 180 cm, and taking vigabatrin 1500 mg q24h; (b) 8 y, weighing 25 kg, measuring 127 cm, and taking vigabatrin 1000 mg q24h.

# Figure S23. The posterior probabilities-concentration curves of zonisamide.


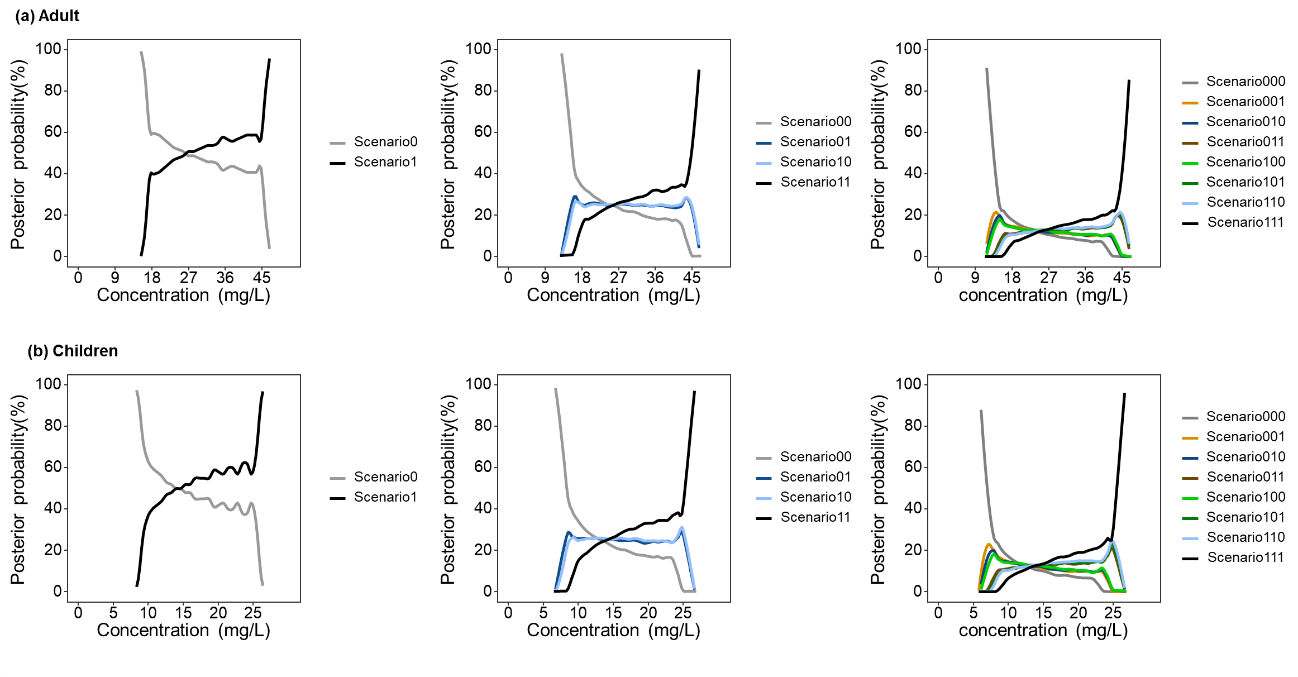


(a) 40 y, weighing 70 kg, measuring 180 cm, and taking zonisamide 200 mg q12h; (b) 8 y, weighing 25 kg, measuring 127 cm, and taking zonisamide 50 mg q12h.

# Figure S24. Effect of renal function on the distinguishability of different dosing scenarios.


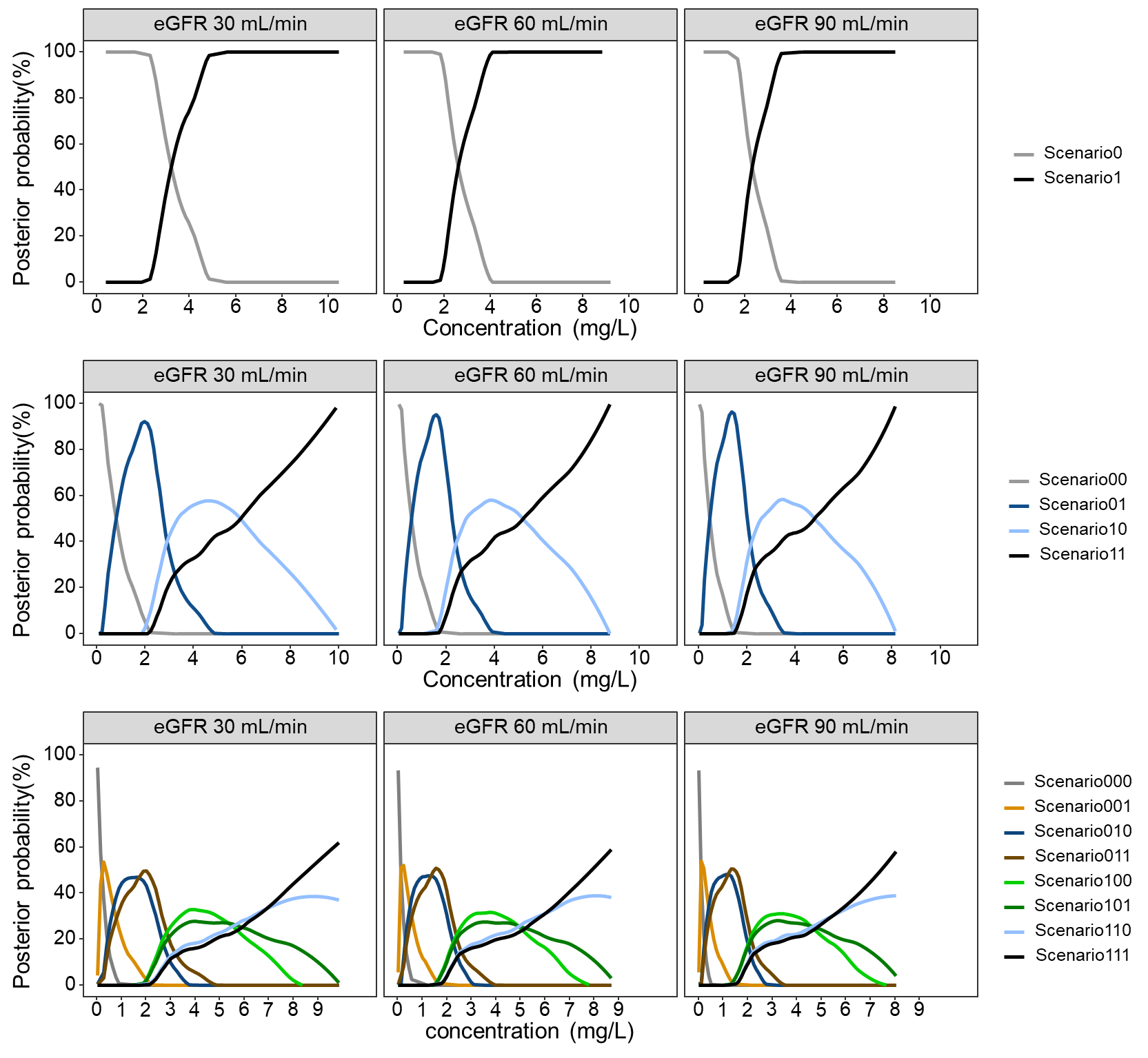


The virtual patient: 40 y, weighing 70 kg, measuring 180 cm, and taking levetiracetam tablet 500 mg q12h.

# Figure S25. Effect of concomitant medicine on the distinguishability of different dosing scenarios.


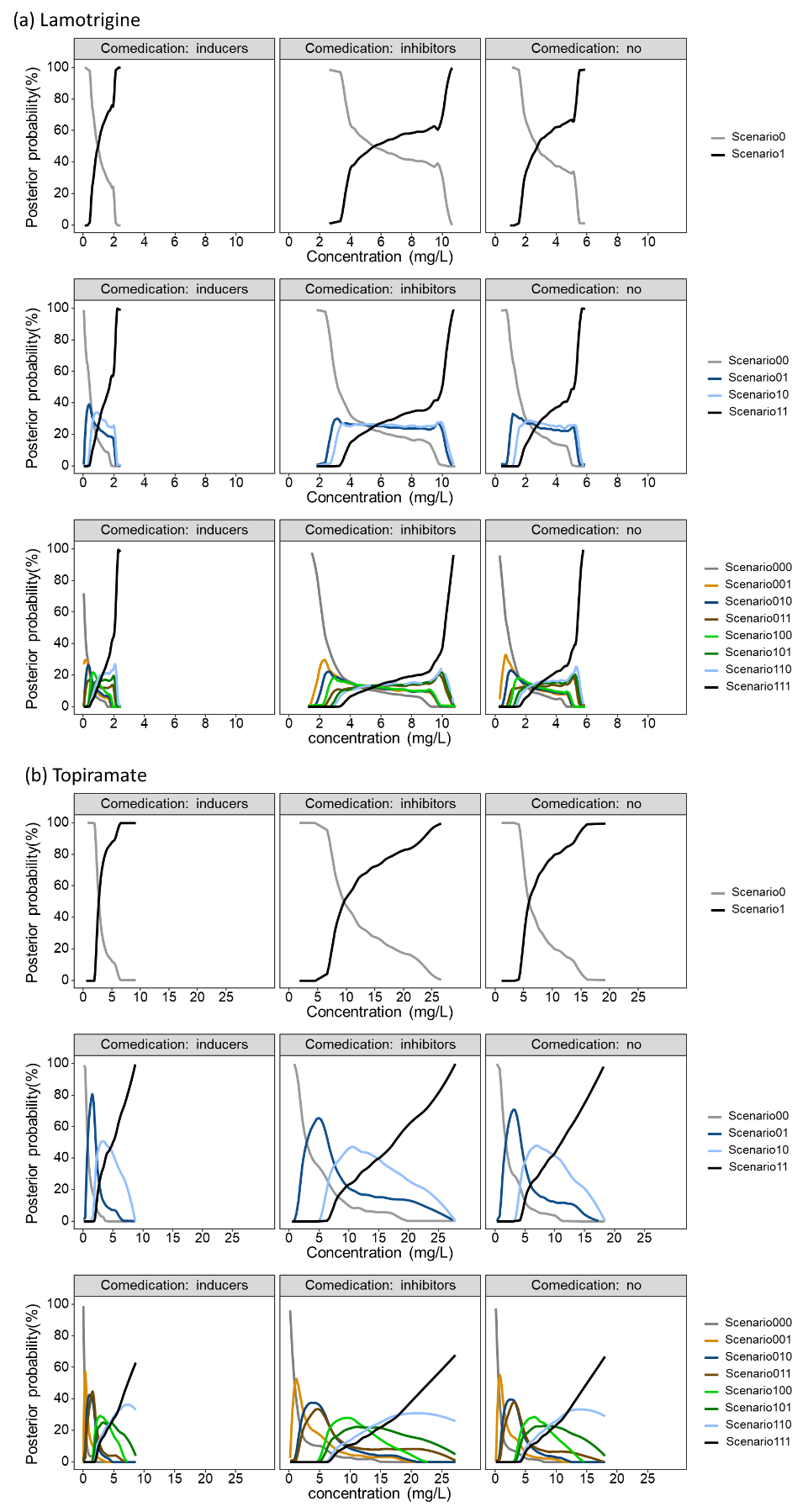


The virtual patient: 40 y, weighing 70 kg, measuring 180 cm, CrCl 90 mL/min, and taking lamotrigine tablet 100 mg q12h, or topiramate 200 mg q12h.

# Figure S26. Effect of sampling time on the distinguishability of dosing behaviors.


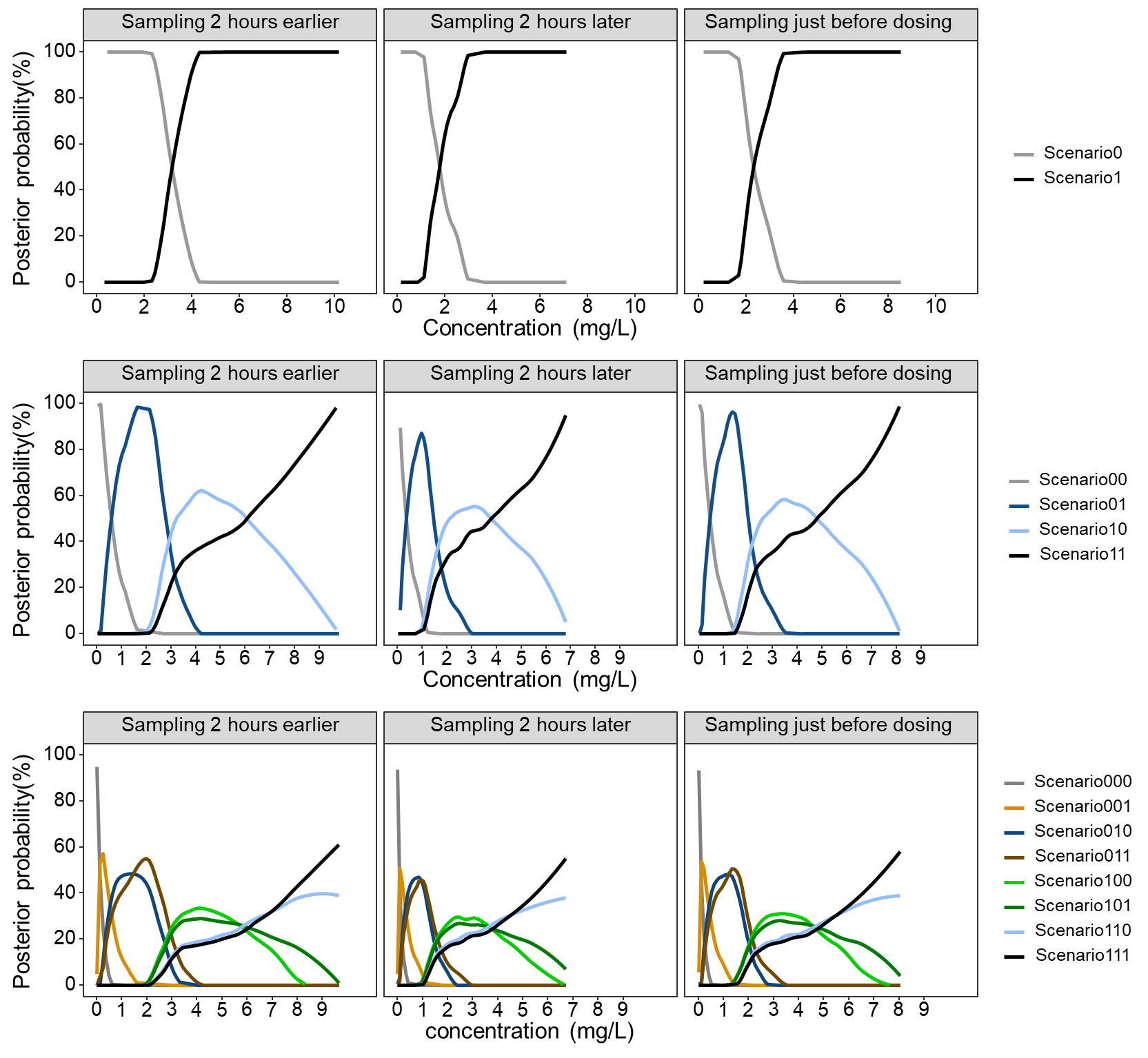
、

The virtual patient: 40 y, weighing 70 kg, measuring 180 cm, and taking levetiracetam tablet 500 mg q12h.

# Figure S27. Effect of prior probability on the distinguishability of dosing behaviors.


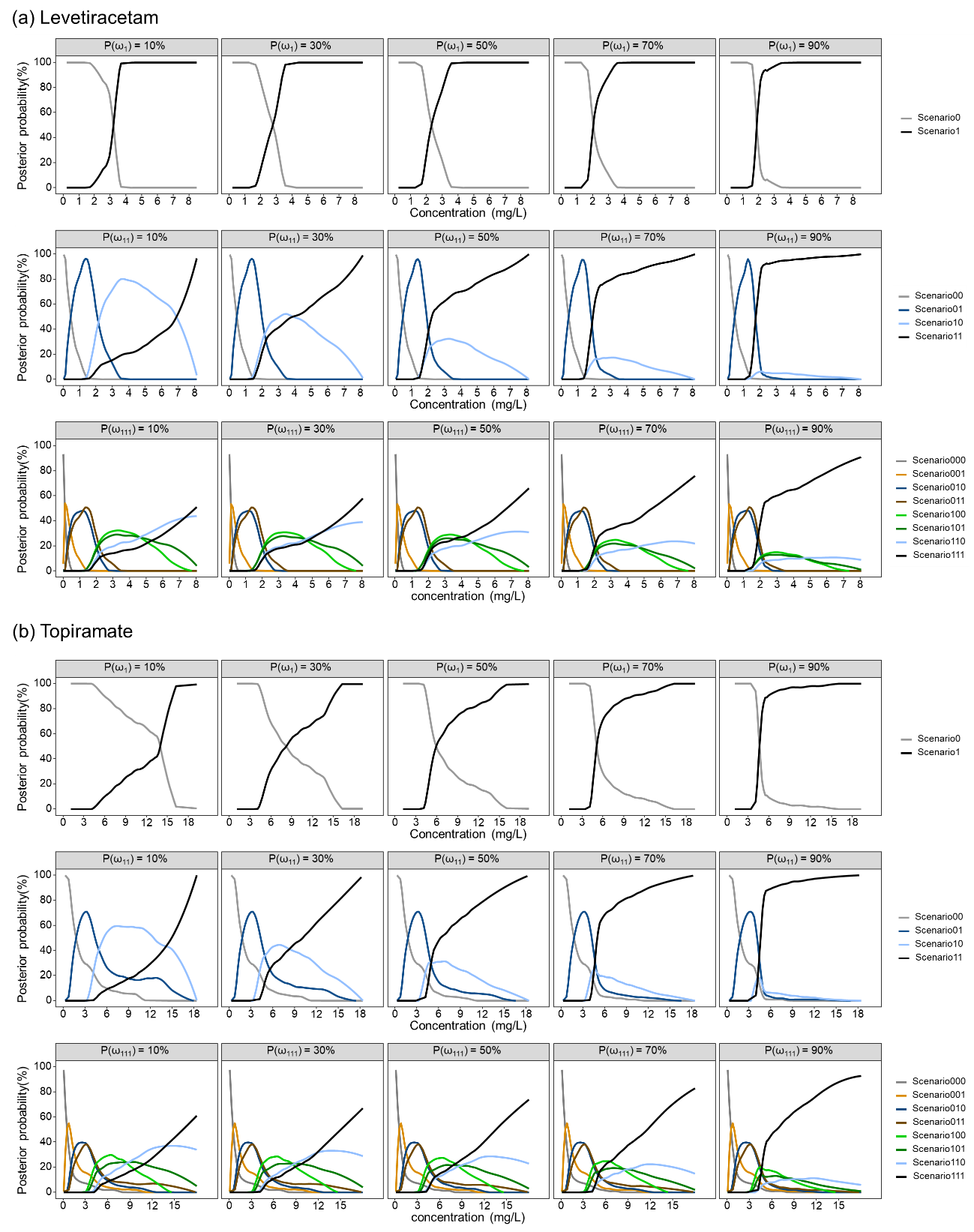


The virtual patient: 40 y, weighing 70 kg, measuring 180 cm, and taking levetiracetam 500 mg q12h, or topiramate 200 mg q12h.

# Figure S28. The effect of prior probability on posterior probability in case 1.


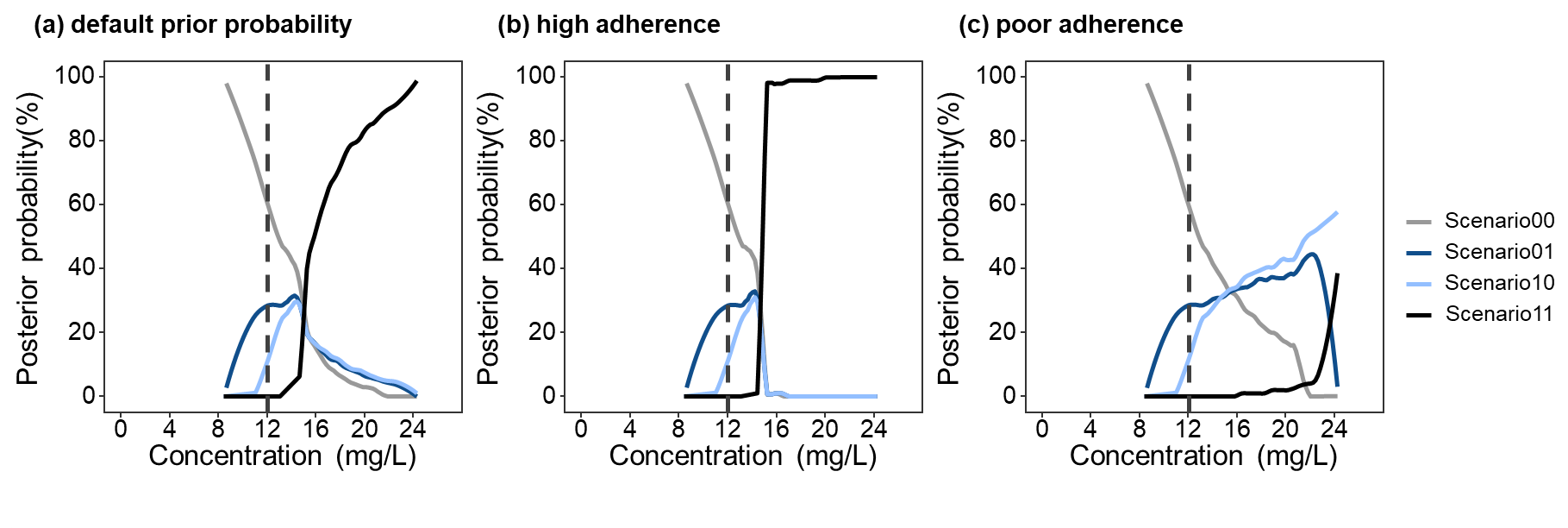


(a) default prior probability, $\omega_{11}$=70%, $\omega_{00}$=$\omega_{01}$=$\omega_{10}$=30%; (b) high adherence, assuming $\omega_{11}$=99%, $\omega_{00}$=$\omega_{01}$=$\omega_{10}$=0.333%; (c) low adherence, assuming $\omega_{11}$=1%, $\omega_{00}$=$\omega_{01}$=$\omega_{10}$=33.33%.

# Figure S29. The effect of residual explained variability on posterior probability in case 1.


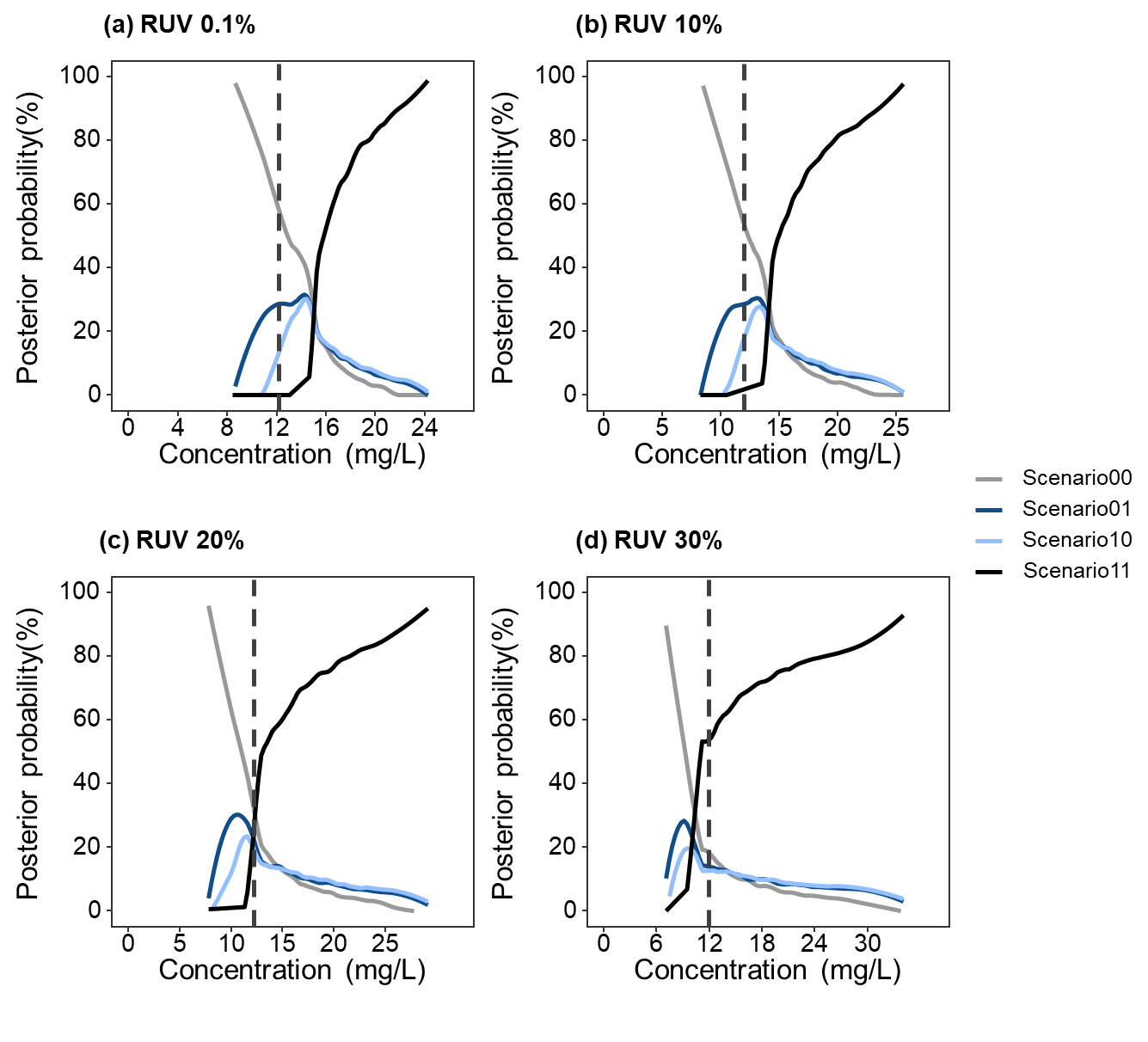


RUV, residual explained variability.

# Figure S30. The effect of prior probability on posterior probability in case 2.


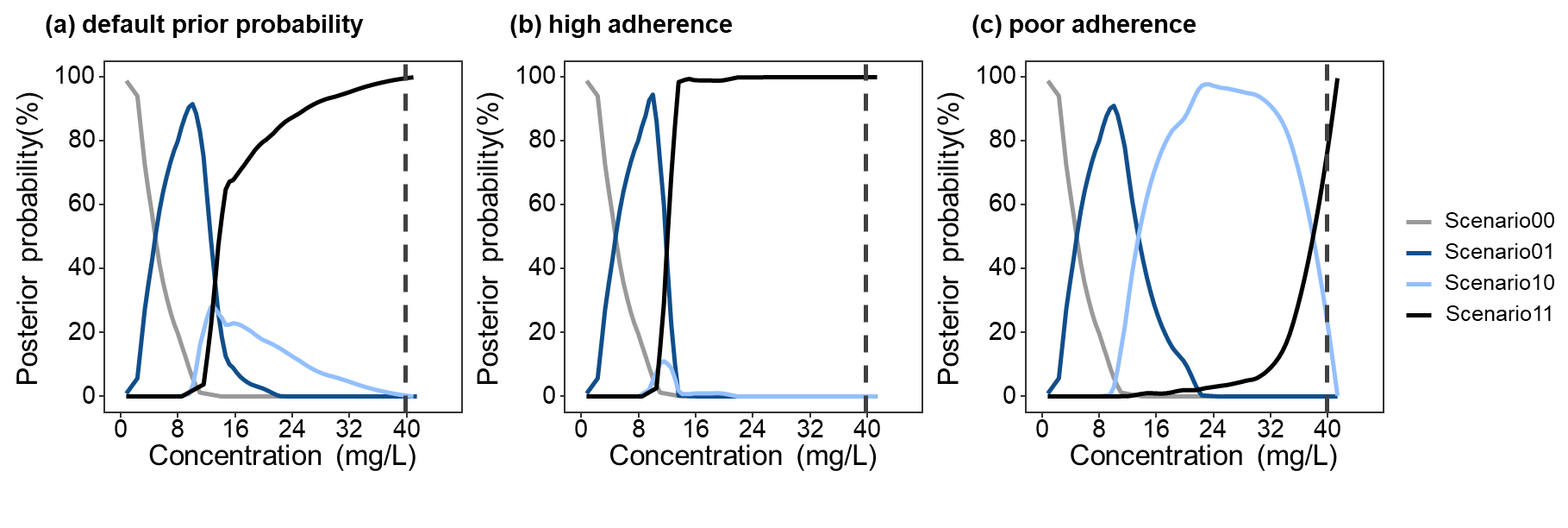


(a) default prior probability, $\omega_{11}$=70%, $\omega_{00}$=$\omega_{01}$=$\omega_{10}$=30%; (b) high adherence, assuming $\omega_{11}$=99%, $\omega_{00}$=$\omega_{01}$=$\omega_{10}$=0.333%; (c) low adherence, assuming $\omega_{11}$=1%, $\omega_{00}$=$\omega_{01}$=$\omega_{10}$=33.33%.

# Figure S31. The effect of residual explained variability on posterior probability in case 2.


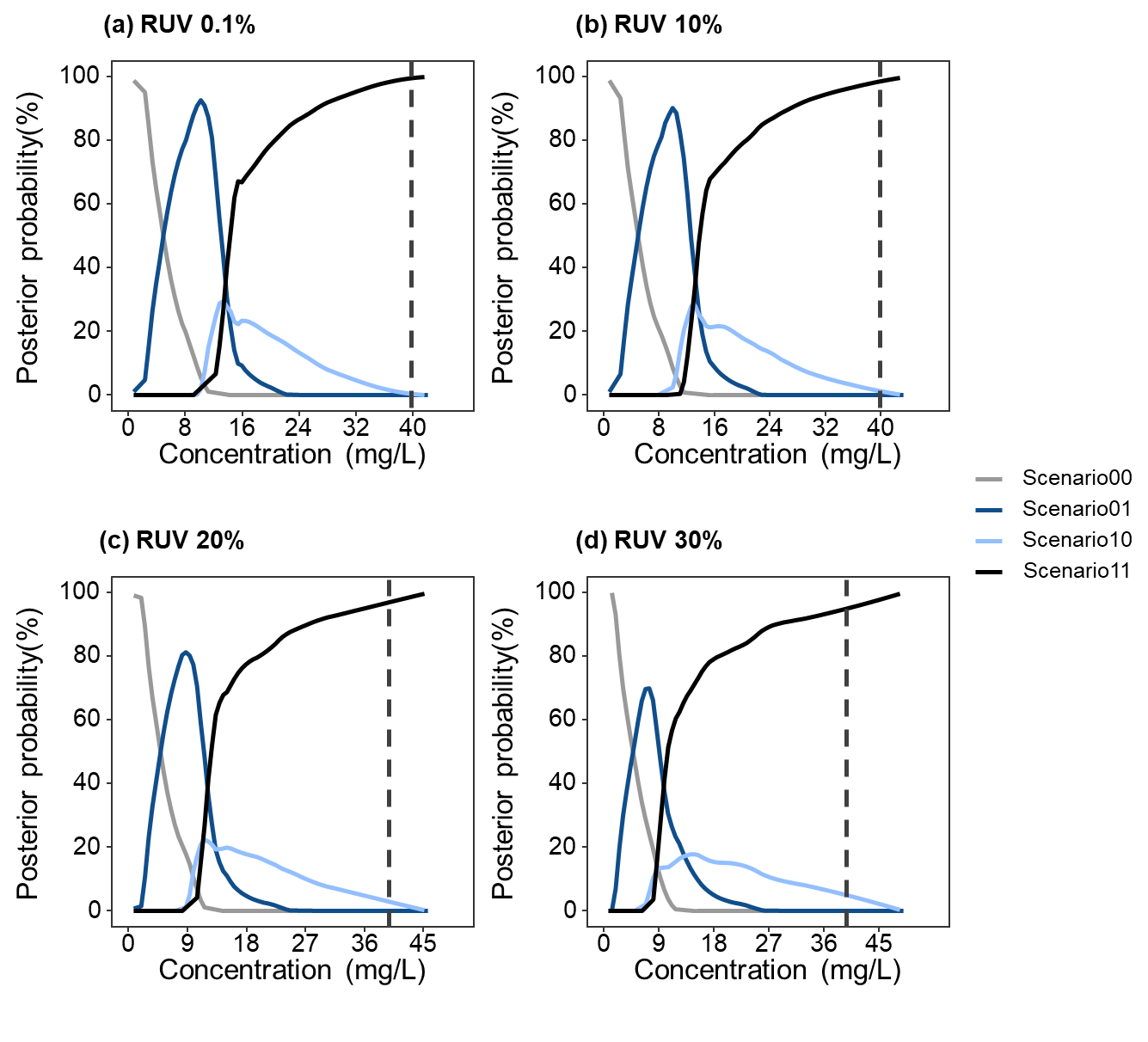


RUV, residual explained variability.

.

# Table S1. The identified population pharmacokinetic studies of antiseizure medications and corresponding dosing regimens.

| **Drug** | **Population** | **Model (year)** | **Dosing regimens** |
| --- | --- | --- | --- |
| Brivaracetam | Children | Schoemaker et al. (2017)[2] | 25 mg q12h |
|  | Adult | Schoemaker et al. (2016)[3] | 50 mg q12h |
| Carbamazepine | Children | Jiao et al. (2004)[4]  Jiao et al. (2004)[4] | 100 mg q12h |
|  | Adult |  | 400 mg q12h |
| Clobazam | Children | Saruwatari et al. (2014)[5]  Saruwatari et al. (2014)[5] | 5 mg q12h |
|  | Adult |  | 10 mg q12h |
| Eslicarbazepine acetate | Children | Sunkaraneni et al. (2018)[6] | 400 mg q24h |
|  | Adult | Gidal et al. (2018)[7] | 800 mg q24h |
| Lacosamide | Children | Winkler et al. (2019)[8] | 100 mg q12h |
|  | Adult | Winkler et al. (2019)[9] | 150 mg q12h |
| Lamotrigine | Children | van Dijkman et al. (2018)[10] | 50 mg q12h  (immediate-release formulation)  100 mg q24h  (extended-release formulation) |
|  | Adult | van Dijkman et al. (2018)[10] | 100 mg q12h  (immediate-release formulation)  200mg q24h  (extended-release formulation) |
|  | Pregnant women | Wang et al. (2021)[11] | 100 mg q12h |
| Levetiracetam | Children | Chhun et al. (2009)[12] | 250 mg q12h |
|  | Adult | Pigeolet et al. (2007)[13] | 500 mg q12h |
|  | Pregnant women | Li et al. (2023)[14] | 500 mg q12h |
| Oxcarbazepine | Children | Lin et al. (2019)[15] | 300 mg q12h |
|  | Adult | Lin et al. (2019)[16] | 600 mg q12h |
| Perampanel | Children | Li et al. (2024)[17] | 4 mg q12h |
|  | Adult | Takenaka et al. (2018)[18] | 8 mg q12h |
| Phenobarbital | Children | Goto et al. (2007)[19]  Goto et al. (2007)[19] | 60 mg q12h |
|  | Adult |  | 60 mg q12h |
| Topiramate | Children | Girgis et al. (2010)[20]  Girgis et al. (2010)[20] | 100 mg q12h |
|  | Adult |  | 200 mg q12h |
| Valproic acid | Children | Ding et al. (2015)[21] | 250 mg q12h  (tablet or syrup)  500 mg q24h  (sustained-release) |
|  | Adult | Teixeira-da-Silva et al. (2022)[22] | 500 mg q12h  (tablet or oral solution)  1000 mg q24h  (modified-release coated tablet) |
| Vigabatrin | Children | Nielsen et al. (2014)[23]  Nielsen et al. (2014)[23] | 1000 mg q24h |
|  | Adult |  | 1500 mg q24h |
| Zonisamide | Children | Okada et al. (2008)[24]  Okada et al. (2008)[24] | 50 mg q12h |
|  | Adult |  | 200 mg q12h |

Children: 8 y, weighting 25 kg, measuring 127 cm, eGFR 90 mL/min; adult: 40 y, weighing 70 kg, measuring 180 cm, eGFR 90 mL/min; pregnant women, 25 y, weighing 70 kg, measuring 160 cm, eGFR 90 mL/min, being 30 weeks pregnant.

# Table S2. Population pharmacokinetic parameter estimates of the identified ASMs studies.

| **Drug (population)** | **Study (Publication year)** | **Brand name** | **Fixed effect parameters** | | **ODE ^[[1]](#endnote-1)^** | **BSV (%)** | **RUV** |
| --- | --- | --- | --- | --- | --- | --- | --- |
| Brivaracetam  (Children) | Schoemaker et al. (2017) [2] | *Briviact* | *Ka* | =1.84 | Equation 1 | 31.9 | 23.4% |
|  |  |  | CL^[[2]](#endnote-2)^ | =3.63×(LBW/50)^0.75^ × 1.479^CBZ^ × 1.408^PB^ × 0.899^VPA^ |  | 22.8 |  |
|  |  |  | V | =47.8×(LBW/50) |  | 16.7 |  |
| Brivaracetam  (Adults) | Schoemaker et al. (2016) [3] | *Briviact* | *Ka* | =1.42 | Equation 1 | 101.2 | 20.7% |
|  |  |  | CL | =3.63×(WT/70)^0.565^ × 1.348^CBZ^ × 1.268 ^PHT^ × 1.239^PB^ |  | 24.7 |  |
|  |  |  | V | =48.1×(WT/70)^0.639^ |  | 30.5 |  |
| Carbamazepine  (Children &  Adults) | Jiao et al. (2004) [4] | *Tegretol, Carbamazepine (Shanghai Huanghe Pharmaceutical Ltd)* | *Ka* | = 1.2 | Equation 1 | / | 14.46%  0.454 mg/L |
|  |  |  | CL | = 0.141 × DD^0.406^ ×WT^0.117^ × 1.23^VPA^ × 1.44^PHT^ × 1.26^PB^ |  | 10.3 |  |
|  |  |  | V | = 72 |  | 42.9 |  |
| Clobazam  (Children &  Adults) | Saruwatari et al. (2014) [5] | *NA* | *Ka* | = 0.0594 | Equation 1 | / | 32.7 % |
|  |  |  | CL^[[3]](#endnote-3)^ | = 0.347 × WT^0.54^ × 0.484^ZNS^ × 1.66^PB^ × 1.93^PHT^ |  | 81.8 |  |
|  |  |  | V | = 13.3 × WT^0.136^ |  | / |  |
| Eslicarbazepine acetate  (Children) | Sunkaraneni et al. (2018) [6] | *Zebinix/Aptiom* | *Ka* | = 0.895 (tablet)  = 4.18 (oral suspension) | Equation 1 | 83.8  / | 23.3 % |
|  |  |  | CL | = 1.69 × (WT/33)^0.75^ × (1 - 0.176 × LEV) × (1 + 0.6626 × PB) |  | 25 |  |
|  |  |  | V | = 32.8 × WT/33 |  | 13.2 |  |
| Eslicarbazepine acetate  (Adults) | Gidal et al. (2018) [7] | *Zebinix/Aptiom* | *Ka* | = 2.34 | Equation 1 | 126.49 | 11.1%  2.3 mg/L |
|  |  |  | CL | = (2.43 + 1.08 × (DCBZ/800)^0.411^ + 1.24 × PBL + 0.0132 × (WT - 70))× (eGFR/115.7)^0.195^ |  | 27.04 |  |
|  |  |  | V | = (61.3 - 9.9 × SEXF + 12 × PBL) × (WT/70)^0.617^ |  | 17.69 |  |

**Table S2. continued**

| **Drug (population)** | **Study (Publication year)** | **Brand name** | **Fixed effect parameters** | | **ODE ^a^** | **BSV (%)** | **RUV** |
| --- | --- | --- | --- | --- | --- | --- | --- |
| Lacosamide (Children) | Winkler et al. (2019) [8] | *Vimpat* | *Ka* | =2.45 | Equation 1 | 55.1 | NA |
|  |  |  | CL | =2.37×(WT/70)^0.624^ × 1.535^IND^ |  | 32.3 |  |
|  |  |  | V | =50.6×(WT/70) |  | 24 |  |
| Lacosamide (Adults) | Winkler et al. (2019) [9] | *Vimpat* | *Ka* | =4.05 | Equation 2 | 70.9 | 50% |
|  |  |  | CL | =2.57×(WT/70)^0.75^× 1.50^CBZ^ × 2.59^PB^× 1.56^PHT^ |  | 23.0 |  |
|  |  |  | V_c_ | =28.2×(WT/70)^0.722^ |  | 35.6 |  |
|  |  |  | V_p_ | =13.1 |  | 55.0 |  |
|  |  |  | Q | =39.1 |  | / |  |
|  |  |  | F | =1.01 |  | 3.8 |  |
|  |  |  | D1 | =0.401 |  | 58.0 |  |
| Lamotrigine  (Adult & Children) | Van Dijkman et al. (2018)[10] | *Lamictal* | *Ka* | = 2.43 (immediate-release formulation)  = 0.087 (extended-release formulation) | Equation 1 | 60.9 (immediate-release formulation)  46(extended-release formulation) | 15.6%  0.236 mg/L |
|  |  |  | CL^[[4]](#endnote-4)^ | = 2.23 ×(WT/70)^0.75^ × 1.765^CBZ^ × 2.29^PHT^ × 0.526^VPA^  = 2.23 ×(WT/70)^0.75^ × 1.765^CBZ^ × 2.29^PHT^ × 0.526^VPA^ × 0.852 (if >65 years) |  | 27.4% |  |
|  |  |  | V | = 1.97 ×(WT/70) × WT |  | 62.6% |  |
| Lamotrigine  (Pregnant women) | Wang et al. (2021)[11] | *NA* | *Ka* | = 1.93 | Equation 1 | / | 30.6 %  0.032 mg/L |
|  |  |  | CL | = 3.30 × (GA/9)^0.347^ × (1 - 0.507 × INH) |  | 37.7 |  |
|  |  |  | V | = 68.8 |  | / |  |

**Table S2. continued**

| **Drug (population)** | **Study (Publication year)** | **Brand name** | **Fixed effect parameters** | | **ODE ^a^** | **BSV (%)** | **RUV** |
| --- | --- | --- | --- | --- | --- | --- | --- |
| Levetiracetam  (Children) | Chhun et al. (2009) [12] | *NA* | *Ka* | = 3.83 | Equation 3 | 117 | 18.90 % |
|  |  |  | Tlag | = 0.283 |  | COR=16.7 |  |
|  |  |  | CL | = 2.47 × (WT/33)^0.89^ |  | 24.3 |  |
|  |  |  | V | = 21.9 × (WT/33)^0.93^ |  | 16.3 |  |
| Levetiracetam  (Adults) | Pigeolet et al. (2007) [13] | *Keppra* | *Ka* | = 2.44 (fed)  = 4.80 (fasted) | Equation 1 | 108 | 27.5 % |
|  |  |  | CL | = 4.02 × (WT/70)^0.268^ × (eGFR/110)^0.122^ × 0.896 ^SEXF^ × 1.09^IND^ × 0.812^VPA^ |  | 19.5 |  |
|  |  |  | V | = 52.7 × (WT/70)^0.952^ |  | 11.8 |  |
| Levetiracetam  (Pregnant women) | Li et al. (2023)[14] | *Keppra* | *K_a_* | = 2.44 | Equation 1 | / | 36.1% |
|  |  |  | CL | = 3.82 × (WT/65)^0.939^ × 1.22 (if pregnant 14~28 weeks)  × 1.15 (if pregnant >28 weeks) |  | 18.9 |  |
|  |  |  | V | = 42 |  | / |  |
| Oxcarbazepine  (Children) | Lin et al. (2019) [15] | *Trileptal* | *Ka* | = 0.83 | Equation 1 | / | 2.992 mg/L |
|  |  |  | CL | = 1.68 × ${(WT/70)}^{(0.624 - (0.233 \times{WT}^{219})/({8.97}^{219} +{WT}^{219}))}$ |  | 11.1 |  |
|  |  |  | V | = 14.7 |  | / |  |
| Oxcarbazepine  (Adults) | Lin et al. (2019) [16] | *Trileptal* | *Ka* | = 0.46 | Equation 1 | / | 24.5 % |
|  |  |  | CL | = 2 × (WT/70)^0.46^ × (eGFR/80)^0.741^ |  | 12.7 |  |
|  |  |  | V | = 102 |  | 58.7 |  |
| Perampanel (Children) | Li et al. (2024) [17] | *Fycompa* | *Ka* | = 1.19 | Equation 1 | / | 17.25% |
|  |  |  | CL | = 0.59 × (WT/70)^0.51^ × (TBIL/5.9)^-0.24^×1.53^OXC^ |  | 44.7 |  |
|  |  |  | V | = 97.27× (WT/70) |  | 97.5 |  |

**Table S2. continued**

| **Drug (population)** | **Study (Publication year)** | **Brand name** | **Fixed effect parameters** | | **ODE ^a^** | **BSV (%)** | **RUV** |
| --- | --- | --- | --- | --- | --- | --- | --- |
| Perampanel (Adults) | Takenaka et al. (2018) [18] | *Fycompa* | *Ka* ^[[5]](#endnote-5)^ | = 1.19 | Equation 1 | / | 8.72% |
|  |  |  | CL^[[6]](#endnote-6)^ | = 0.668 × (ALT/17)^-0.0901^×0.822^SEXF^×0.908^Asian^  ×2.95^CBZ^×1.99^OXC/PHT^ ×1.21^TOP/PB^ |  | 43.5 |  |
|  |  |  | V | = 43.5 |  | 51.5 |  |
| Phenobarbital  (Children &  Adults) | Goto et al. (2007) [19] | *NA* | *Ka* | = 2 | Equation 1 | / | 3.49 mg/L |
|  |  |  | CL ^c^ | = 0.223 × (WT/40)^0.21^ × 0.68^VPA^ × 0.85^PHT^ |  | 17.3 |  |
|  |  |  | V | = 14.78 |  | 171.2 |  |
| Topiramate  (Children &  Adults) | Girgis et al. (2010) [20] | *Topamax* | *Ka* | = 0.105 | Equation 4 | 22.34 | 25.46 %  0.1797 mg/L |
|  |  |  | *K_23_* | = 0.577 |  | / |  |
|  |  |  | *K_32_* | = 0.0586 |  | / |  |
|  |  |  | CL | = 1.21 × (WT/69.9)^0.453^ × $e^{(-0.00306 \times(Age - 31.4))}$× 0.686^VPA^ × 1.94^IND^ |  | 27.28 |  |
|  |  |  | V | = 4.61 × (WT/69.9)^1.14^ |  | 116.2 |  |
| Valproic acid  (Children) | Ding et al. (2015) [21] | *Depakine, Baoqing, Depakine* | *Ka* | = 2.64 (syrup, Depakine^®^)  = 1.57 (conventional tablet, Baoqing^®^)  = 0.46 (sustained-release tablet, Depakine^®^) | Equation 1 | / | 13.3 mg/L |
|  |  |  | CL | = 0.3 × 1.43^CBZ^ × ${(WT/70)}^{(0.791-0.096\times\mathrm{Age}^{8.63}/({0.802}^{8.63} + \mathrm{Age}^{8.63}))}$  × (1 + 2.8 × DD^1.68^/(37.4^1.68^ + DD^1.68^)) |  | 19.5% |  |
|  |  |  | V | = 22.2 × (WT/70) |  | / |  |
| Valproic acid  (Adults) | Teixeira-da-Silva et al. (2022) [22] | *NA* | *Ka* | = 2.64 (oral solution)  = 0.78 (gastro-resistant tablet)  = 0.38 (modified-release coated tablet) | Equation 1 | / | 8.5 mg/L |
|  |  |  | CL | = 0.1 × (WT/60)^0.7^ × DD^0.2^ × 1.36^CBZ^ × 1.25^PHT^ × 1.11^PB^ |  | 18 |  |
|  |  |  | V | = 0.14 × WT |  | / |  |

**Table S2. continued**

| **Drug (population)** | **Study (Publication year)** | **Brand name** | **Fixed effect parameters** | | **ODE ^a^** | **BSV (%)** | **RUV** |
| --- | --- | --- | --- | --- | --- | --- | --- |
| Vigabatrin (Children & Adult) | Nielsen et al. (2014) [23] ^[[7]](#endnote-7)^ | *Sabril* | CL | =6.52×(eGFR/100)^0.538^×0.525 (for children aged 3-16 years) | Equation 5 | / | 93.3% ^[[8]](#endnote-8)^ |
|  |  |  | V_c_ | = 23.9 × (WT/70)^0.406^ |  | 18.2% |  |
|  |  |  | k | =CL/V_c_ |  | 14.5% |  |
|  |  |  | V_p_ | = 32.3 × (WT/70)^1.03^ |  | / |  |
|  |  |  | Q | = 3.59 × (WT/70)^0.692^ |  | / |  |
|  |  |  | ktr | = 11.4 × (Age/28)^0.3^ |  | 22.9% |  |
|  |  |  | F | = 1 |  | / |  |
|  |  |  | n | = 5 |  | / |  |
| Zonisamide  (Children &  Adults) | Okada et al. (2008) [24] | *NA* | *Ka* | = 2 | Equation 1 | / | 22.4 % |
|  |  |  | CL ^c^ | = 1.065 × (WT/44)^0.77^ × DD^-0.17^ × 1.24^CBZ^ × 1.28^PHT^ × 1.29^PB^ |  | 27.6 |  |
|  |  |  | V | = 1.23 × WT |  | / |  |

*ALT* Alanine Aminotransferase (IU/L); *AGE* age of patients (year); *CBZ* carbamazepine; *CL* apparent clearance (L/h); *COR* covariance between CL and V; *D1* duration for zero-order absorption (h); *DCBZ* Carbamazepine daily dose (mg); *DD* daily dose (mg/day); *eGFR* estimated glomerular filtration rate (mL/min/1.73m^2^), *F* absorption rate; *GA* gestational age (weeks); *IND* enzyme-inducing antiseizure medications; *INH*, enzyme-inhibiting antiseizure medications; *k* elimination rate constant (h^-1^); *K_a_* absorption rate constant (h^-1^); *KM* Michaelis constant; *K_23_* inter-compartment rate constants from central to peripheral compartment (h^-1^); *K_32_* inter-compartment rate constants from peripheral to central compartment (h^-1^); *ktr* transit rate constant (h^-1^); *LBW* lean body weight (kg); *LEV* levetiracetam; *n* number of transit compartments; *ODE, ordinary differential equations*; *OXC* oxcarbazepine; *PB* phenobarbital; *PBL* Phenobarbital-like AEDs including phenytoin and primidone; *PHT* phenytoin; *Q* inter-compartment clearance between the central and peripheral compartment; *SEXF* 1 for female and 0 for male; *TBIL* total bilirubin; *Tlag* absorption lag time (h); *TOP* topiramate; *V* apparent volume of distribution (L); *V_c_* apparent volume of distribution of the central compartment (L); *VM* maximum reaction velocity; *V_p_* apparent volume of distribution of the peripheral compartment (L); *VPA* valproate acid; *WT* body weight (kg); *ZNS* zonisamide.

a **Equation 1**: d(A1)/dt=-*Ka*×A(1); d(A2)/dt= *Ka*×A(1)-CL/V×A(2) ; **Equation 2**: When t<D1: d(A1)/dt=-F×Dose/D1; d(A2)/dt= F×Dose/D1-CL/V_c_×A(2)-Q/V_c_×A(2)+ Q/V_p_×A(3); d(A3)/dt= Q/V_c_×A(2)-Q/V_p_×A(3). When t≥D1: d(A1)/dt=-*Ka*×A(1); d(A2)/dt= *Ka*×A(1)- CL/V_c_×A(2)-Q/V_c_×A(2)+ Q/V_p_×A(3); d(A3)/dt= Q/V_c_×A(2)-Q/V_p_×A(3); **Equation 3**: When t<Tlag: d(A1)/dt=0; d(A2)/dt= -CL/V×A(2); when t≥Tlag: d(A1)/dt=-*Ka*×A(1); d(A2)/dt= *Ka*×A(1)-CL/V×A(2); **Equation 4**: d(A1)/dt=-*Ka*×A(1); d(A2)/dt= *Ka*×A(1)-*K_23_*×A(2)-CL/V×A(2)+*K_32_*×A(3); d(A3)/dt= *K_23_*×A(2)-*K_32_*×A(3); **Equation 5**: d(A1)/dt=F×Dose×ktr- ktr×A(1); d(A2)/dt=ktr×A(1)-ktr×A(2); d(A3)/dt=ktr×A(2)-ktr×A(3); d(A4)/dt=ktr×A(3)-ktr×A(4); d(A5)/dt=ktr×A(4)-ktr×A(5); d(A6)/dt= ktr×A(5)-CL/V_c_×A(6)-Q/V_c_×A(6)+ Q/V_p_×A(7); d(A7)/dt= Q/V_c_×A(6)-Q/V_p_×A(7).

b For males: LBW=1.1×WT-0.0128×(WT/HT)^2^, for females: LBW=1.07×WT-0.0148×(WT/HT) ^2^, where WT is weight (kg), HT is height (m).

c The original equation contains the effect of genotype, and the equation used in the dashboard has been normalized according to gene frequency reported in the original literature.

d CL for patients aged <2 years is not included in the dashboard.

e The *Ka* was fixed from another study conducted in children [17].

f Asian=0, if Japanese, Chinese and non-Asian; Asian=1, if Asians except for Japanese and Chinese. In the dashboard, the CL for Japanese, Chinese and non-Asian was defaulted.

g The model is simplified as a two-compartment model with first-order absorption and elimination when developing the dashboard to shorten the calculation time.

h The residual unexplained variability in study 118 was applied.

# Reference

1. Li ZR, Wang CY, Lin WW, Chen YT, Liu XQ, Jiao Z. Handling Delayed or Missed Dose of Antiseizure Medications: A Model-Informed Individual Remedial Dosing. Neurology. Feb 28 2023;100:e921-e31. [doi:10.1212/WNL.0000000000201604] [Medline:36450606]

2. Schoemaker R, Wade JR, Stockis A. Brivaracetam population pharmacokinetics in children with epilepsy aged 1 month to 16 years. Eur J Clin Pharmacol. Jun 2017;73:727-33. [doi:10.1007/s00228-017-2230-6] [Medline:28280887]

3. Schoemaker R, Wade JR, Stockis A. Brivaracetam population pharmacokinetics and exposure-response modeling in adult subjects with partial-onset seizures. J Clin Pharmacol. Dec 2016;56:1591-602. [doi:10.1002/jcph.761] [Medline:27146213]

4. Jiao Z, Shi XJ, Zhao ZG, Zhong MK. Population pharmacokinetic modeling of steady state clearance of carbamazepine and its epoxide metabolite from sparse routine clinical data. J Clin Pharm Ther. Jun 2004;29:247-56. [doi:10.1111/j.1365-2710.2004.00557.x] [Medline:15153086]

5. Saruwatari J, Ogusu N, Shimomasuda M, Nakashima H, Seo T, Tanikawa K, et al. Effects of CYP2C19 and P450 oxidoreductase polymorphisms on the population pharmacokinetics of clobazam and N-desmethylclobazam in japanese patients with epilepsy. Ther Drug Monit. Jun 2014;36:302-9. [doi:10.1097/FTD.0000000000000015] [Medline:24345815]

6. Sunkaraneni S, Ludwig E, Fiedler-Kelly J, Hopkins S, Galluppi G, Blum D. Modeling and simulations to support dose selection for eslicarbazepine acetate therapy in pediatric patients with partial-onset seizures. J Pharmacokinet Pharmacodyn. Aug 2018;45:649-58. [doi:10.1007/s10928-018-9596-7] [Medline:29948795]

7. Gidal BE, Jacobson MP, Ben-Menachem E, Carreno M, Blum D, Soares-da-Silva P, et al. Exposure-safety and efficacy response relationships and population pharmacokinetics of eslicarbazepine acetate. Acta Neurol Scand. Sep 2018;138:203-11. [doi:10.1111/ane.12950] [Medline:29732549]

8. Winkler J, Schoemaker R, Stockis A. Population pharmacokinetics of adjunctive lacosamide in pediatric patients with epilepsy. J Clin Pharmacol. Apr 2019;59:541-7. [doi:10.1002/jcph.1340] [Medline:30427550]

9. Winkler J, Schoemaker R, Stockis A. Modeling and simulation for the evaluation of dose adaptation rules of intravenous lacosamide in children. Epilepsy Res. Jan 2019;149:13-6. [doi:10.1016/j.eplepsyres.2018.10.011] [Medline:30415109]

10. van Dijkman SC, de Jager NCB, Rauwe WM, Danhof M, Della Pasqua O. Effect of age-related factors on the pharmacokinetics of lamotrigine and potential implications for maintenance dose optimisation in future clinical trials. Clin Pharmacokinet. Aug 2018;57:1039-53. [doi:10.1007/s40262-017-0614-5] [Medline:29363050]

11. Wang ML, Tao YY, Sun XY, Guo Y, Wang ZY, Cao YF, et al. Estrogen profile- and pharmacogenetics-based lamotrigine dosing regimen optimization: Recommendations for pregnant women with epilepsy. Pharmacol Res. Jul 2021;169:105610. [doi:10.1016/j.phrs.2021.105610] [Medline:33857625]

12. Chhun S, Jullien V, Rey E, Dulac O, Chiron C, Pons G. Population pharmacokinetics of levetiracetam and dosing recommendation in children with epilepsy. Epilepsia. May 2009;50:1150-7. [doi:10.1111/j.1528-1167.2008.01974.x] [Medline:19175400]

13. Pigeolet E, Jacqmin P, Sargentini-Maier ML, Stockis A. Population pharmacokinetics of levetiracetam in Japanese and Western adults. Clin Pharmacokinet. 2007;46:503-12. [doi:10.2165/00003088-200746060-00004] [Medline:17518509]

14. Li Y, Wang ML, Guo Y, Cao YF, Zhao MM, Zhao LM. Population pharmacokinetics and dosing regimen optimization of levetiracetam in epilepsy during pregnancy. Br J Clin Pharmacol. Mar 2023;89:1152-61. [doi:10.1111/bcp.15572] [Medline:36260320]

15. Lin WW, Li XW, Jiao Z, Zhang J, Rao X, Zeng DY, et al. Population pharmacokinetics of oxcarbazepine active metabolite in Chinese paediatric epilepsy patients and its application in individualised dosage regimens. Eur J Clin Pharmacol. Mar 2019;75:381-92. [doi:10.1007/s00228-018-2600-8] [Medline:30456415]

16. Lin WW, Wang CL, Jiao Z, Yu XL, Zhang J, Zhang WB, et al. Glomerular filtration rate is a major predictor of clearance of oxcarbazepine active metabolite in adult Chinese epileptic patients: a population pharmacokinetic analysis. Ther Drug Monit. Oct 2019;41:665-73. [doi:10.1097/FTD.0000000000000644] [Medline:31033858]

17. Li S, Yi J, Tuo Y, Nie G, Wang J, Wang Y, et al. Population pharmacokinetics and dosing optimization of perampanel in children with epilepsy: A real-world study. Epilepsia. Jun 2024;65:1687-97. [doi:10.1111/epi.17954] [Medline:38572689]

18. Takenaka O, Ferry J, Saeki K, Laurenza A. Pharmacokinetic/pharmacodynamic analysis of adjunctive perampanel in subjects with partial-onset seizures. Acta Neurol Scand. Apr 2018;137:400-8. [doi:10.1111/ane.12874] [Medline:29171002]

19. Goto S, Seo T, Murata T, Nakada N, Ueda N, Ishitsu T, et al. Population estimation of the effects of cytochrome P450 2C9 and 2C19 polymorphisms on phenobarbital clearance in Japanese. Ther Drug Monit. Feb 2007;29:118-21. [doi:10.1097/FTD.0b013e318030def0] [Medline:17304159]

20. Girgis IG, Nandy P, Nye JS, Ford L, Mohanty S, Wang S, et al. Pharmacokinetic-pharmacodynamic assessment of topiramate dosing regimens for children with epilepsy 2 to <10 years of age. Epilepsia. Oct 2010;51:1954-62. [doi:10.1111/j.1528-1167.2010.02598.x] [Medline:20880232]

21. Ding J, Wang Y, Lin W, Wang C, Zhao L, Li X, et al. A population pharmacokinetic model of valproic acid in pediatric patients with epilepsy: a non-linear pharmacokinetic model based on protein-binding saturation. Clin Pharmacokinet. Mar 2015;54:305-17. [doi:10.1007/s40262-014-0212-8] [Medline:25388986]

22. Teixeira-da-Silva P, Perez-Blanco JS, Santos-Buelga D, Otero MJ, Garcia MJ. Population pharmacokinetics of valproic acid in pediatric and adult caucasian patients. Pharmaceutics. Apr 7 2022;14:811. [doi:10.3390/pharmaceutics14040811] [Medline:35456645]

23. Nielsen JC, Kowalski KG, Karim A, Patel M, Wesche DL, Tolbert D. Population pharmacokinetics analysis of vigabatrin in adults and children with epilepsy and children with infantile spasms. Clin Pharmacokinet. Nov 2014;53:1019-31. [doi:10.1007/s40262-014-0172-z] [Medline:25172554]

24. Okada Y, Seo T, Ishitsu T, Wanibuchi A, Hashimoto N, Higa Y, et al. Population estimation regarding the effects of cytochrome P450 2C19 and 3A5 polymorphisms on zonisamide clearance. Ther Drug Monit. Aug 2008;30:540-3. [doi:10.1097/FTD.0b013e31817d842a] [Medline:18641551]

1. ) ; **Equation 2**: When t<D1: d(A1)/dt=-F×Dose/D1; d(A2)/dt= F×Dose/D1-CL/V_c_×A(2)-Q/V_c_×A(2)+ Q/V_p_×A(3); d(A3)/dt= Q/V_c_×A(2)-Q/V_p_×A(3). When t≥D1: d(A1)/dt=-*Ka*×A(1); d(A2)/dt= *Ka*×A(1)- CL/V_c_×A(2)-Q/V_c_×A(2)+ Q/V_p_×A(3); d(A3)/dt= Q/V_c_×A(2)-Q/V_p_×A(3); **Equation 3**: When t<Tlag: d(A1)/dt=0; d(A2)/dt= -CL/V×A(2); when t≥Tlag: d(A1)/dt=-*Ka*×A(1); d(A2)/dt= *Ka*×A(1)-CL/V×A(2); **Equation 4**: d(A1)/dt=-*Ka*×A(1); d(A2)/dt= *Ka*×A(1)-*K_23_*×A(2)-CL/V×A(2)+*K_32_*×A(3); d(A3)/dt= *K_23_*×A(2)-*K_32_*×A(3); **Equation 5**: d(A1)/dt=F×Dose×ktr- ktr×A(1); d(A2)/dt=ktr×A(1)-ktr×A(2); d(A3)/dt=ktr×A(2)-ktr×A(3); d(A4)/dt=ktr×A(3)-ktr×A(4); d(A5)/dt=ktr×A(4)-ktr×A(5); d(A6)/dt= ktr×A(5)-CL/V_c_×A(6)-Q/V_c_×A(6)+ Q/V_p_×A(7); d(A7)/dt= Q/V_c_×A(6)-Q/V_p_×A(7). [↑](#endnote-ref-1)
2. For males: LBW=1.1×WT-0.0128×(WT/HT/100)^2^, for females: LBW=1.07×WT-0.0148×(WT/HT/100) ^2^, where WT is weight (kg), HT is height (cm). [↑](#endnote-ref-2)
3. The original equation contains the effect of genotype, and the equation used in the dashboard has been normalized according to gene frequency reported in the original literature. [↑](#endnote-ref-3)
4. CL for patients aged <2 years is not included in the dashboard. [↑](#endnote-ref-4)
5. The *Ka* was fixed from another study conducted in children [17]. [↑](#endnote-ref-5)
6. Asian=0, if Japanese, Chinese and non-Asian; Asian=1, if Asians except for Japanese and Chinese. In the dashboard, the CL for Japanese, Chinese and non-Asian was defaulted. [↑](#endnote-ref-6)
7. The model is simplified as a two-compartment model with first-order absorption and elimination when developing the dashboard to shorten the calculation time. [↑](#endnote-ref-7)
8. The residual unexplained variability in study 118 was applied. [↑](#endnote-ref-8)
